# Composition and Antimicrobial Activity of *Ilex* Leaves Water Extracts

**DOI:** 10.3390/molecules26247442

**Published:** 2021-12-08

**Authors:** Emil Paluch, Piotr Okińczyc, Anna Zwyrzykowska-Wodzińska, Jakub Szperlik, Barbara Żarowska, Anna Duda-Madej, Przemysław Bąbelewski, Maciej Włodarczyk, Wioleta Wojtasik, Robert Kupczyński, Antoni Szumny

**Affiliations:** 1 Department of Microbiology, Faculty of Medicine, Wroclaw Medical University, Chałubińskiego 4, 50-376 Wrocław, Poland; emil.paluch@umed.wroc.pl (E.P.); anna.duda-madej@umw.edu.pl (A.D.-M.); 2 Department of Pharmacognosy and Herbal Medicines, Wroclaw Medical University, Borowska 211a, 50-556 Wrocław, Poland; maciej.wlodarczyk@umw.edu.pl; 3 Department of Environment Hygiene and Animal Welfare, Wrocław University of Environmental and Life Sciences, Chełmońskiego 38C, 51-630 Wrocław, Poland; anna.zwyrzykowska@upwr.edu.pl (A.Z.-W.); robert.kupczynski@upwr.edu.pl (R.K.); 4 Laboratory of Tissue Culture, Botanical Garden, Faculty of Biological Sciences, University of Wrocław, Sienkiewicza 23, 50-525 Wrocław, Poland; jakub.szperlik@uwr.edu.pl; 5 Department of Biotechnology and Food Microbiology, Wrocław University of Environmental and Life Sciences, Chełmońskiego 37, 51-630 Wrocław, Poland; barbara.zarowska@upwr.edu.pl; 6 Department of Horticulture, Wrocław University of Environmental and Life Sciences, Grunwaldzki 24A, 50-375 Wrocław, Poland; 7 Faculty of Biotechnology, University of Wrocław, Przybyszewskiego 63/77, 51-148 Wrocław, Poland; wioleta.wojtasik@uwr.edu.pl; 8 Department of Chemistry, Wrocław University of Environmental and Life Sciences, Norwida 25, 50-375 Wrocław, Poland; antoni.szumny@upwr.edu.pl

**Keywords:** *Ilex*, antimicrobial activity, biofilm, HPLC, DAD, MS/MS, polyphenols, yerba mate

## Abstract

Plants from the *Ilex* genus are known for properties such as antimicrobial and anti-inflammatory activity, can act as antiobesity agents and thus can be helpful in medicine. Some holly species, such as *Ilex paraguariensis* (widely known in the form of popular beverage: yerba mate), have been investigated, while others have been partially researched or remain unknown. Therefore, we performed qualitative and quantitative phytochemical analyses and screened antimicrobial properties of lesser-studied species (*I. aquifolium* L., *I. aquifolium* ‘Argentea Marginata’ and *I.* × *meserveae* ‘Blue Angel’). *I. paraguariensis* was used as a standard species for comparison purposes. Investigations were performed on water extracts due to their expected activity and composition. Antimicrobial research included evaluating minimal inhibitory, bactericidal (*Staphylococcus aureus* and *Escherichia coli*) and fungicidal concentration (*Candida albicans*, *Alternaria alternata*, *Fusarium oxysporum*, and *Aspergillus niger*) of extracts. The influence of the extracts on the production, eradication, and viability of bacterial biofilms was also analysed. It was established that *Ilex paraguariensis* possesses the richest profile of hydroxycinnamic acids derivatives in terms of component concentration and diversity. *Ilex* spp., especially *I.* × *meserveae*, contain a slightly higher amount of flavonoids and more different flavonoid derivatives than *I. paraguariensis*. However, the strongest antibacterial activity was shown by *I. aquifolium* L. and its cultivar ‘Argentea Marginata’ in terms of minimal inhibitory, bactericidal and fungicidal concentration, and biofilm assays. Extracts from both species significantly reduced the biofilm viability of *S. aureus* as well, which may be of use in the production of multicomponent lavaseptics, antiseptics, diuretics (supporting urinary tract infection therapy) and, due to their action on fungi, additives to growth media for specific fungi. The significant content of saponins enables *Ilex* extracts to be used as natural emulsifiers, for example, in cosmetics. Moreover, relatively high chlorogenic acid and rutin content may suggest use of *Ilex* spp. to treat obesity, digestive problems, in chemoprevention, and as preservatives in the food industry.

## 1. Introduction

Over the past several years, there has been increased interest in searching for a natural approach to food production. This is partly due to the common belief that natural substances are less toxic than artificially synthesised preservatives. Therefore products perceived as natural are bought and consumed more readily, e.g., consuming fruits, herbs and vegetables could improve public health [1].

Yerba mate, an infusion prepared from the leaves of *Ilex paraguariensis* A. St. Hilaire (*Aquifoliaceae*), is widely consumed worldwide. In producer countries (e.g., Argentina, Brazil), mate production is an important branch of agriculture and has a special status in the economy. The leaves of *I. paraguariensis* used for preparing the beverage has become a valuable source of bioactive substances known to have therapeutic efficacy in treating or preventing the development of arthritis, inflammatory diseases, cardiovascular diseases, haemorrhoids, headache, hepatic disorders, and obesity [2,3]. It is known that different yerba mate beverages are used in South American folk medicine, for example, by the Guarani people [4].

Biologically active compounds found in *I. paraguariensis* may differ depending on extraction methods, genetic and environmental variability as well as harvest time [5,6]. Generally, these extracts contain polyphenols, purines (caffeine, theobromine), vitamins A, B, C, and E, tannins, hydroxycinnamic acids esters of quinic acids (mainly caffeoylquinic acids [5,7,8,9,10]) as well as some triterpene saponins derived from ursolic acid [3,10,11].

Recent inquiries have revealed the antimicrobial potential of some *Ilex* species extracts, whose spectrum of activity encompasses Gram-positive and Gram-negative bacteria and fungi [12,13,14]. Overall, *I. paraguariensis* extracts were usually more active against Gram-positive than Gram-negative bacteria species [12,13,14]. It is noteworthy though that activity was retained against Gram-negative pathogens responsible for bacterial diarrhoeas such as *Escherichia coli* O157:H7 [13], *Salmonella enteritidis* and *Listeria monocytogenes* [12]. Therefore, as a popular drink, Yerba mate may be used to treat diarrhoea as a supporting agent with other drugs. It is especially noteworthy that *Ilex* species extracts were also active against drug-resistant strains [13]. Apart from bacteria, *Ilex* exhibits antifungal activity. For example, Haraguchi et al. [15] has shown that *I. integra* is a potent antifungal agent against *Candida albicans*. Usually, the antimicrobial activity of *Ilex* species was associated with polyphenols (mainly chlorogenic acids and their derivates [12]) and triterpenoids [15].

Among all of the *Ilex* genus species, research is generally focused to a large extent on *I. paraguariensis* due to its importance in the food industry as a source of drinks containing caffeine. In contrast, other *Ilex* species composition and biological properties usually remain partially investigated or unexplored at all. To our best knowledge, among selected species, only *I. paraguariensis* has been the subject of intensive phytochemical and pharmacological studies; therefore, we focused on other holly species easily cultivable in European temperate climate: *I. aquifolium* L., *Ilex aquifolium* ‘Argentea Marginata’, and *Ilex* × *meserveae* ‘Blue Angel’. We decided to use these species and cultivars because of their easy growth in a temperate climate, availability and low environmental requirements. Moreover, these plants have been cultivated in Poland for many years as ornamental plants. Overall, these characteristics may make them promising crop species in the future.

A significant amount of hydroxycinnamic acids esters of quinic acid in these species (previous research [9]), as well as reported antimicrobial activity of *I. paraguariensis* water extract [14,16] and antibiofilm properties of *I. guayusa* [17], make holly species candidates for antibacterial research. Moreover, the presence of saponins in the investigated species [18] justifies using water as an extractant. Usually, water is not considered a proper solvent due to the high sugar content in water extracts; sugars can stimulate microbial growth. However, we supposed that at the same time, emulsifying properties of saponins should increase the solubility of expected antimicrobial agents (hydroxycinnamic acids esters of quinic acid) in water. Moreover, this solution may be classified as ‘green chemistry’ and support saving the environment from ‘hard chemicals’.

In summary, our aims were screening research of antimicrobial properties as well as qualitative and quantitative analysis of *I. aquifolium* L., *Ilex aquifolium* ‘Argentea Marginata’, and *Ilex* × *meserveae* ‘Blue Angel’ leaves water extracts. *I. paraguariensis* was used as standard species for comparison purposes due to the most exhaustive research of yerba mate. Antimicrobial tests included the evaluation of microorganism growth curves, MIC (minimal inhibitory concentration) and MBC (minimal bactericidal concentration) or MFC (minimal fungicidal concentration). Moreover, antibiofilm properties of extracts against *Staphylococcus aureus* and *Escherichia coli* were also investigated. The antibiofilm panel is composed of assays of inhibition of bacterial adhesion, biofilm production, viability and eradication. *S. aureus* and *E. coli* were chosen for biofilm tests according to their model status as Gram-positive and Gram-negative bacteria, respectively.

The highlights of our research include:

- UHPLC-DAD-MS/MS qualitative and quantitative analysis of *I. aquifolium* L., *I. aquifolium* ‘Argentea Marginata’ and *I.* × *meserveae* ‘Blue Angel’ water extracts focused on polyphenols components.
- Antibacterial and antifungal activity (evaluation of MIC, MBC/MFC and bacteria and fungi growth curves) of *Ilex* spp water extracts (*I. aquifolium* L., *I. aquifolium* ‘Argentea Marginata’ and *I.* × *meserveae* ‘Blue Angel’).
- Influence of *Ilex* spp. water extracts (*I. aquifolium* L., *I. aquifolium* ‘Argentea Marginata’ and *I.* × *meserveae* ‘Blue Angel’) on bacterial adhesion, production, viability and eradication of *Staphylococcus aureus* and *Escherichia coli* biofilm.

In the future, purified extracts of investigated *Ilex* species (or their preparations) may be sources of antibacterial agents, as well part of different multicomponent antimicrobial preparations. However, this requires screening evaluation of basic antimicrobial properties and phytochemical composition.

## 2. Results

### 2.1. UHPLC-DAD-MS/MS Phytochemical Profiles and Quantification of Ilex Species Water Extracts

#### 2.1.1. Qualitative Analysis of *Ilex* Species Water Extracts

**Figure A1:**
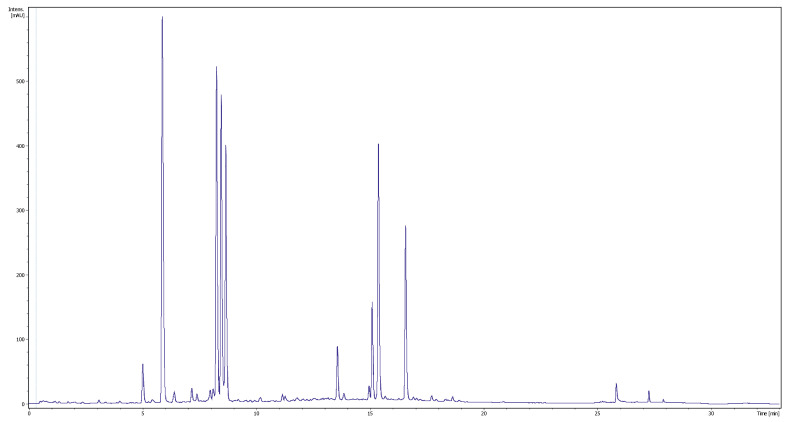
*Ilex paraguariensis* A.St.-Hil. leaves water extract, UV chromatogram, 280 nm.

**Figure A2:**
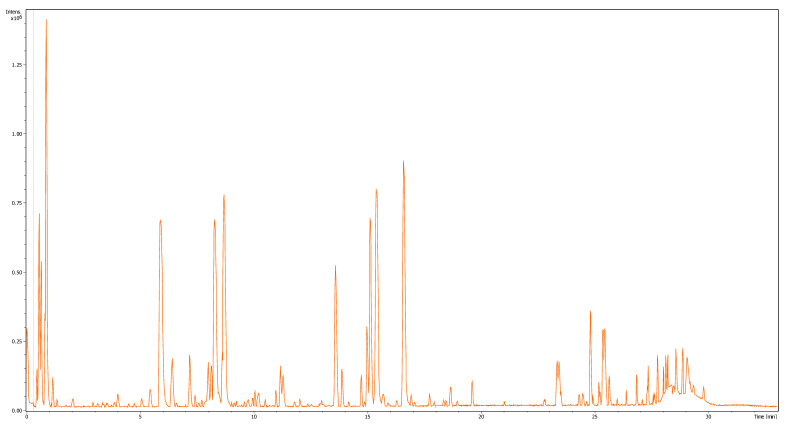
*Ilex paraguariensis* A.St.-Hil. leaves water extract, basic peak chromatogram, negative mode.

**Figure A3:**
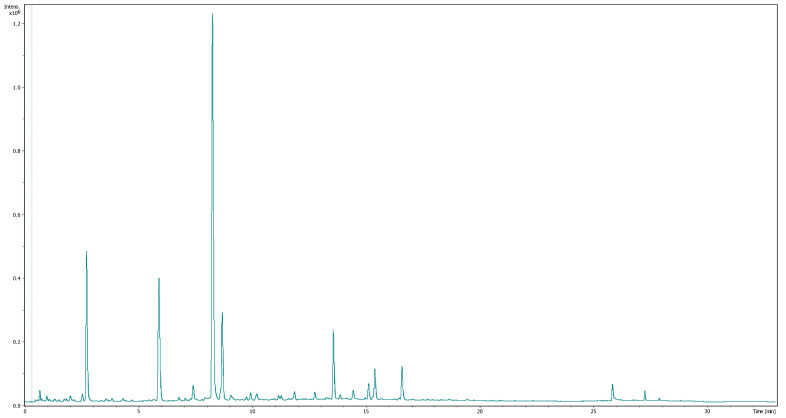
*Ilex aquifolium* L. leaves water extract, UV chromatogram, 280 nm.

**Figure A4:**
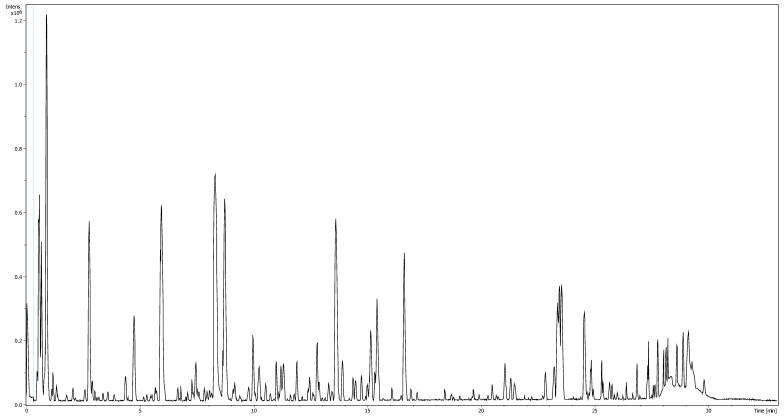
*Ilex aquifolium* L. leaves water extract, basic peak chromatogram, negative mode.

**Figure A5:**
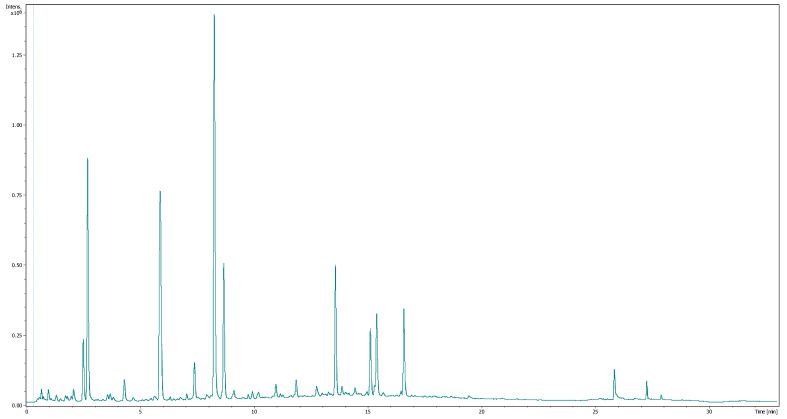
*Ilex aquifolium* ‘Argentea Marginata’ leaves water extract, UV chromatogram, 280 nm.

**Figure A6:**
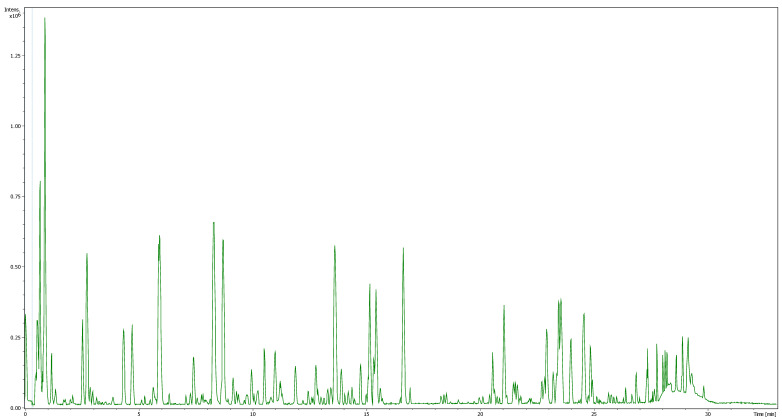
*Ilex aquifolium* ‘Argentea Marginata’, basic peak chromatogram, negative mode.

**Figure A7:**
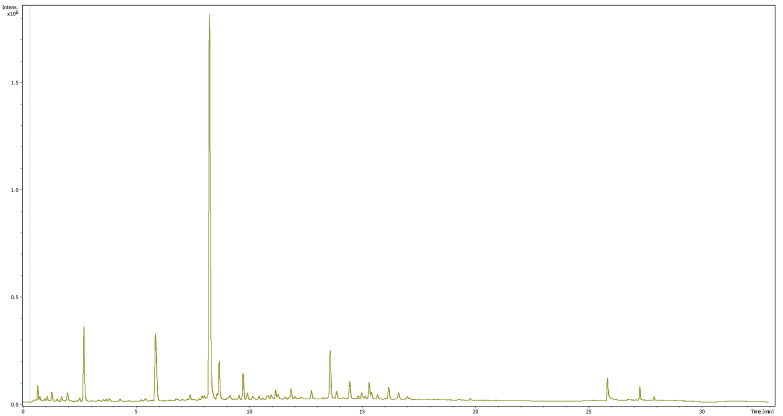
*Ilex* × *meserveae* ‘Blue Angel’ leaves water extract, UV chromatogram, 280 nm.

**Figure A8:**
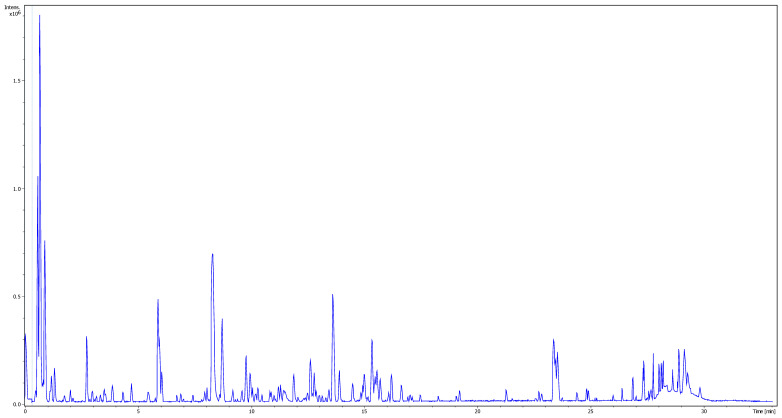
*Ilex* × *meserveae* ‘Blue Angel’, basic peak chromatogram, negative mode.

Results are presented in Table 1 (identification of components), Table 2 (presence of components) and Table 3 (quantification of phenolic acid derivates and flavonoids). In our research, MS and MS/MS analyses were supported by an analysis of UV absorption spectra. This approach allowed us to successfully identify most phenolic components by comparing mass and UV spectra with known data on standards and literature. In addition, some isomers were determined according to elution order in analyses performed in similar conditions recorded in the literature. The error between the calculated and experimental *m/z* for deprotonated ions was lower than 5.0 ppm. Detailed data on UHPLC-DAD-MS/MS qualitative analysis are available in Appendix A (Table A1 and Figure A1, Figure A2, Figure A3, Figure A4, Figure A5, Figure A6, Figure A7 and Figure A8).

UHPLC-DAD-MS/MS revealed the presence of 66 non-saponin components. These substances were divided into several groups: hydroxycinnamic acids and their derivates (28 compounds), flavonoid glycosides (24 compounds), methylxanthines (two compounds) and others (19 compounds). Extracts were also rich in saponins, but these were analysed previously and, therefore, not in our focus [18]. Components were detectable in MS negative mode (NEG), except for methylxanthines which were ionised only in the positive mode (POS). Therefore, ESI-NEG was used as a basic ionisation method.

The hydroxycinnamic acids derivatives group were dominated by caffeic acid esters (mainly mono- and dicaffeoyl quinic acids). Among monoesters the main components were chlorogenic (3-caffeoylquinic acid; 3-CQA), neochlorogenic (5-CQA) and cryptochlorogenic acid (4-CQA) while dicaffeoylesters included 3,5-dicaffeoylquinic acid (3,5-CQA), 4,5-dicaffeoylquinic acid (4,5-CQA) and 3,4-dicaffeoylquinic acid (3,4-CQA). Apart from mono and dicaffeoyl esters, samples contained monoferuloyl, mono-*p*-coumaroyl and caffeoylferuloylquinic acids and some other phenols such as caffeoylglucose. Generally, the greatest variety of phenolic acid derivatives was observed for *I. paraguariensis* and the poorest for *I.* × *meserveae*.

The second considerable group of compounds, flavonoids, were mainly glycosides of quercetin, kaempferol and isorhamnetin. It was found that analysed *Ilex* taxons possess very similar profiles of flavonoids. In all of the samples, rutin is by far the most abundant of flavonoids. Among all investigated samples, *I.* × *meserveae* exhibited the most diverse profile of flavonoids.

The third group, methylxanthines, included caffeine, theobromine and trace of theophylline, present only in *I. paraguariensis* leaves.

The last group, named ‘other components’, included mainly unidentified substances, phenols and components which were not derivatives of hydroxycinnamic acids (e.g., cyanogenic glucosides). Components in this group were usually tentatively identified due to the lack of accurate UV and (or) MS/MS data. Moreover, in some cases, MS/MS generally agreed with the literature, but UV did not. These components may have similar skeletons to those proposed, but the substituents are probably different.

#### 2.1.2. Quantitative UHPLC-DAD Analysis of *Ilex* Species Water Extracts

Results of the analysis are presented in Table 3. Phenolic acid derivatives were calculated as mg of chlorogenic or *p*-coumaric acid equivalents per gram of dry mass (mg ChE g^−1^ and mg pCE g^−1^, respectively) and flavonoids as rutin equivalents per gram of dry mass (mg RuE g^−1^). The calculated standard deviation for each measurement was lower than 5%. The highest concentration of polyphenols was observed for *I. paraguariensis*, *I. aquifolium*, *I. aquifolium* ‘Argentea Marginata’ and *I.* × *meserveae*, respectively.

In our work, *I. paraguariensis* had a large amount of hydroxycinnamic acids derivates (81.31 ± 1.81 mg ChAE g^−1^). The rest of the investigated *Ilex* leaves possessed significantly lower phenolic acid derivates per gram (from 42.71 in *I. aquifolium* to 22.96 mg ChE g^−1^ in *I.* × *meserveae*). Generally, *I. paraguariensis* contained more hydroxycinnamic derivates, mainly mono and dicaffeoyl quinic acids, than European *Ilex* species. Moreover, various caffeoylquinic acids dominated in different samples. *I. paraguariensis* contained a similar amount of neochlorogenic and chlorogenic acids, while the other species contained mostly chlorogenic acid; the concentration of neochlorogenic acid was about four times lower. However, in the case of dicaffeoylquinic acids, 3,5-dicaffeoylquinic acid was the most abundant in this group for all samples.

For flavonoids, *I. paraguariensis* contained the lowest (3.62 mg RuE g^−1^) amount and *I.* × *meserveae* ‘Blue Angel’ the highest (8.26 mg RuE g^−1^). The highest amount of single flavonoid was observed for rutin in all samples: from 2.21 in *I.* × *meserveae* to 4.37 mg RuE g^−1^ in *I. aquifolium* ‘Argentea Marginata’. The remaining flavonoids were found below 1.00 mg RuE g^−1^ except for quercetin-rutinoside (1.13 mg in *I.* × *meserveae*). Generally, differences in amounts of flavonoids between samples were lower than in the case of hydroxycinnamic acids derivatives. Usually, those were caused by the absence of some flavonoid derivatives in most cases.

### 2.2. Evaluation of the Antimicrobial Activity of Ilex spp. Water Extracts

Tested extracts of *Ilex* spp. were subjected to multistage microbiological tests to determine their activity against selected strains of bacteria and fungi. Tests were performed to determine their antimicrobial efficacy against both free cells forms and biofilms.

#### 2.2.1. Evaluation of Bacterial and Fungal Growth Curves

**Figure 1:**
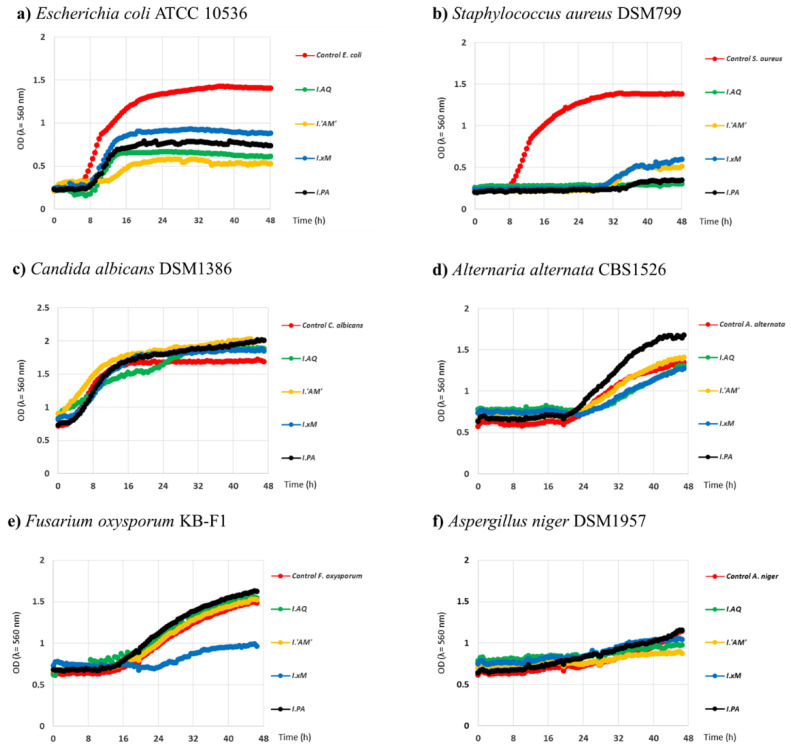
Growth curves of selected strains of bacteria and fungi under the influence of water extracts from Ilex spp in time 48 h. **Legend:** Legend: *I.PA*—*I. paraguariensis*; *I.AQ*—*I. aquifolium*; *I.’AM’*—*I. aquifolium* ‘Argentea Marginata’; *I.×M*—*I.* × *meserveae* ‘Blue Angel’.

The evaluation results of bacterial and fungal growth curves are presented in Figure 1.

As shown in Figure 1, all *Ilex* extracts decreased the growth rate of *S. aureus* and *E. coli.* A more pronounced effect was observed against *S. aureus* than *E. coli.* The growth curves for the mentioned strains of bacteria under the influence of the tested water extracts showed a weakened growth expressed as optical density below 1 OD and for *S*. *aureus* a growth delay of between 8-16 h (*I. aquifolium* ‘Argentea Marginata’ and *I. paraguariensis*) in relation to control. This strongly suggests that the extracts may be bacteriostatic. The growth curves under the influence of *Ilex* spp. extracts for the fungal strains were very similar to the controls. Only the activity of *I.* × *meserveae* ‘Blue Angel’ extract was different, where a strong inhibition of the growth of the *F*. *oxysporum* strain was observed. There were differences between the activity of the extracts as well. The most significant effect was observed for *I. aquifolium*; *I. paraguariensis*, and *I. aquifolium* ‘Argentea Marginata’.

In the case of fungi, the results were variable. For most cases, extracts exhibited very low growth stimulation or had no effect. The greatest growth stimulation was observed for *I. aquifolium* ‘Argentea Marginata’ used against *C. albicans*, especially at the beginning. After 48h, the remaining extracts showed similar effects. Clear stimulation of growth was also exhibited by *I. paraguariensis* against *A. alternata*. Among all tested extracts, clear growth inhibition of fungi was found only for *I.* × *meserveae* ‘Blue Angel’ against *F. oxysporum*. Its growth was inhibited for 24 h and then only slightly increased.

#### 2.2.2. Analysis of Minimum Inhibitory Concentration (MIC) and Fungicidal and Bactericidal Concentrations (MFC/MBC)

Results of the analysis of antimicrobial screening are presented in Table 4. Measurements of MIC and MBC demonstrated that the most potent antibacterial effect was observed against *S. aureus* and *E. coli* for *I. aquifolium* and *I. aquifolium* ‘Argentea Marginata’ extracts and were in the range of 0.26 mg mL^−1^–1.02 mg mL^−1^. Bactericidal concentration was obtained for concentrations ranging from 0.26 mg mL^−1^ to 2.06 mg mL^−1^. For selected fungal strains, the strongest activity was observed for *C. albicans* and *A. alternata* reaching 1.02 mg mL^−1^, for the abovementioned extracts. Extracts tested on other fungal strains were less potent, often reaching values above 2.06 mg mL^−1^.

#### 2.2.3. Activity of Tested Extracts against Biofilm

The next stage of the research was to determine the activity of the tested *Ilex* spp. extracts against the biofilm of selected bacterial strains. In the first stage of the study, the extracts were most active against *E. coli* and *S. aureus* for which bacteriostatic activity was previously relatively low. This part of our study determined both the influence of the extracts on the adhesion of bacteria and the ability to form biofilm under flow conditions with medium on a glass surface and under conditions without medium flow in 96-well titration plates. Due to the presence of chlorogenic acids and their derivatives, as well as saponins, *Ilex* species are promising candidates for agents used against bacterial biofilm formation.

##### Influence of *Ilex* spp. Extracts on Adhesion Process and Biofilm Formation of Bacterial Strains in the Microfluidic Flow System

**Figure 2:**
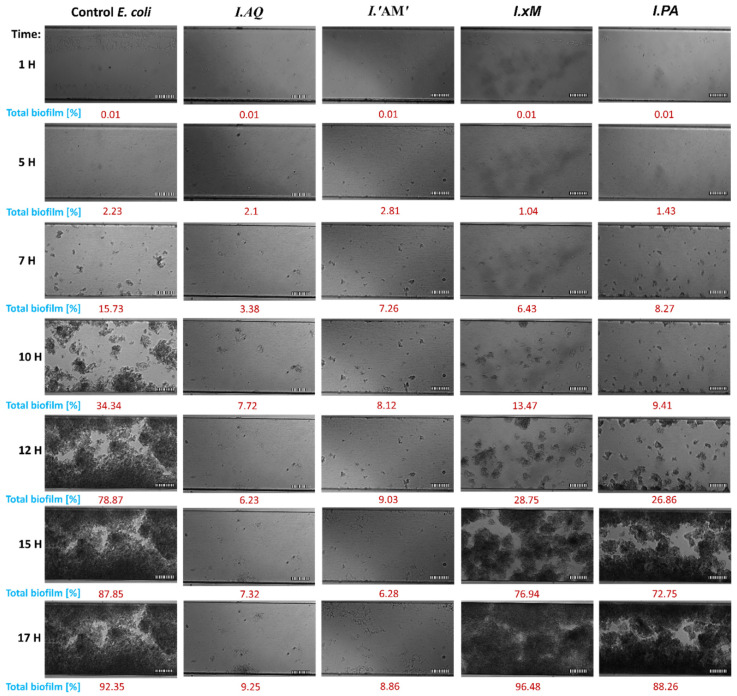
Imaging of the channel cross section with *E*. *coli* biofilms during 17 h of incubation at flow conditions (0.5 dyn cm^2^). Biofilms were formed by bacteria treated with *Ilex* spp. water extracts (1/2 MIC). Biofilms were visualised using differential interference contrast (DIC); Control—untreated bacteria; scale bar = 100 µm. Legend: *I.PA*—*I. paraguariensis*; *I.AQ*—*I. aquifolium*; *I.’AM’*—*I. aquifolium* ‘Argentea Marginata’; *I.×M*—*I.* × *meserveae* ‘Blue Angel’.

**Figure 3:**
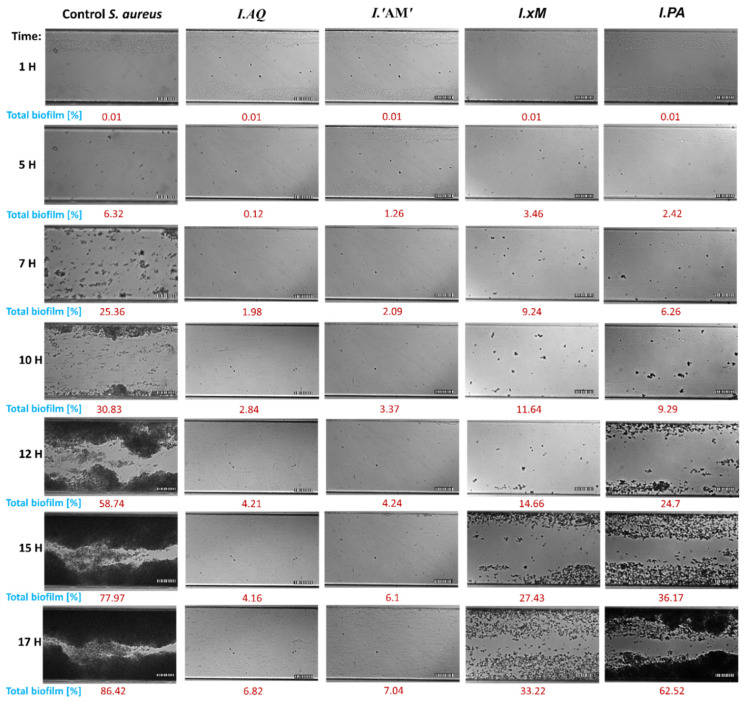
Imaging of the channel cross section with of *S*. *aureus* biofilms after 17 h of incubation at flow conditions (0.5 dyn cm^2^). Biofilms were formed by bacteria treated with water *Ilex* spp. extracts (1/2 MIC). Biofilms visualized using differential interference contrast (DIC); Control—untreated bacteria; scale bar = 100 µm. Legend: *I.PA*—*I. paraguariensis*; *I.AQ*—*I. aquifolium*; *I.’AM’*—*I. aquifolium* ‘Argentea Marginata’; *I.×M*—*I.* × *meserveae* ‘Blue Angel’.

This study determined the effect of the tested extracts on the adhesion process and the ability to create a biofilm of *E. coli* and *S. aureus* in the microfluidic flow system of medium during 17 h long incubation. Therefore, growth curves from the experiment, which established 1/2 MIC values of *Ilex* spp. were used. The experiment duration was adjusted to the optimal flow conditions in the test channels (the biofilm may clog the channels preventing the flow of medium if left to grow for a sufficiently long time). The results are shown in Figure 2 and Figure 3.

A detailed analysis of the results of the influence of the tested extracts on the adhesion and biofilm formation of *E. coli* showed a substantial delay in the bacterial adhesion process. Generally, the critical point of the experiments was reached at 12 h when the control exhibited 78.87% of total biofilm formation. At this critical point, *I. aquifolium* ‘Argentea Marginata’, *I. aquifolium*, *I. paraguariensis* and *I.* × *meserveae* showed 6.23, 9.03, 26.86 and 28.75% of total biofilm formation, respectively. However, the strongest effect was observed at the 15 h mark of the experiment. At this point, *I. aquifolium* ‘Argentea Marginata’ exhibited 6.28% and *I. aquifolium* 7.32% of total biofilm formation in comparison to 87.85% in control and above 72% in the remaining samples. Strong inhibition of biofilm formation caused by *I. aquifolium* ‘Argentea Marginata’ and *I. aquifolium* slightly decreased by the end of the experiment, when they exhibited 8.86% and 9.25% of total biofilm formation, respectively. At this time, the remaining samples showed no effect on biofilm formation or, at best, a very weak inhibition.

The results of extract activity against *S. aureus* were very similar. A strong influence on the inhibition of the adhesion process up to the 12 h mark was observed. The amount of biofilm formed under the influence of the extracts was also significantly reduced compared to the control (86.42%). The strongest activity was, again, demonstrated by *I. aquifolium* and *I. aquifolium* ‘Argentea Marginata’ extracts, inhibiting biofilm formation up to 10% during the experiment.

##### Influence of Extracts on Biofilm Formation

**Figure 4:**
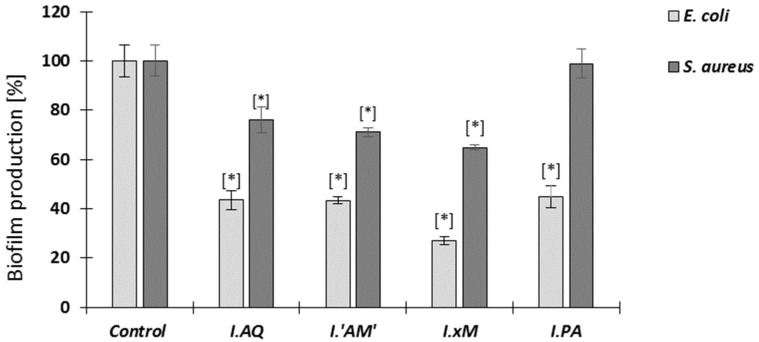
Influence of *Ilex* spp. extracts (at 1/2 MIC concentration) on biofilm production *E*. *coli* and *S*. *aureus* after 24 h. The control is bacteria without extracts; mean ± SD, *n* = 3; * statistically different from the control *p* < 0.05. Legend: *I.PA*—*I. paraguariensis*; *I.AQ*—*I. aquifolium*; *I.’AM’*—*I. aquifolium* ‘Argentea Marginata’; *I.×M*—*I.* × *meserveae* ‘Blue Angel’.

In this experiment, the influence of *Ilex* spp. on *E. coli* and *S. aureus* biofilm production on polystyrene surfaces over a 24 h period was observed. Results are shown in Figure 4.

An analysis of the results of the influence on the biofilm production of the tested extracts showed significant inhibition of the tested bacterial strains. It was significantly potent in the case of *E. coli*, where a steep reduction of the amount of biofilm was noticed (from 55% to less than 40% compared to the control). For *S. aureus*, the inhibition of biofilm production by most extracts was also noticeable, ranging from 70% to 80%; however, it was not as effective as in the case of *E. coli.* It is also worth mentioning that the extract of *I. paraguariensis* showed no effect on the production of *S. aureus* biofilm.

##### Influence of Extracts on Biofilm Eradication

**Figure 5:**
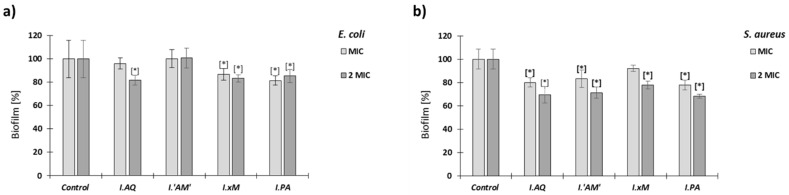
Influence of water *Ilex* extracts (MIC and 2 MIC) on biofilm eradication *E*. *coli* (**a**) and *S*. *aureus* (**b**) after 24 h. The control is biofilm not treated with extracts; mean ± SD, *n* = 3; * statistically different from the control *p* < 0.05. Legend: *I.PA*—*I. paraguariensis*; *I.AQ*—*I. aquifolium*; *I.’AM’*—*I. aquifolium* ‘Argentea Marginata’; *I.×M*—*I.* × *meserveae* ‘Blue Angel’.

In this experiment, the tested extracts’ ability to eradicate 24 h biofilm of *E. coli* and *S. aureus* was determined at two concentrations corresponding to the MIC and 2 MIC. The results are shown in Figure 5.

Eradication of the tested bacterial biofilms for all tested extracts was quite low and similar to each other both in the concentration of MIC and 2 MIC. The results showed that eradication of *S. aureus* biofilm by *Ilex* spp. extracts was stronger than *E. coli* biofilm (with 70—80% biofilm preservation compared to the control).

##### Biofilm Viability Measured by Fluorescence Microscopy after the Action of *Ilex* ssp. Extracts

**Figure 6:**
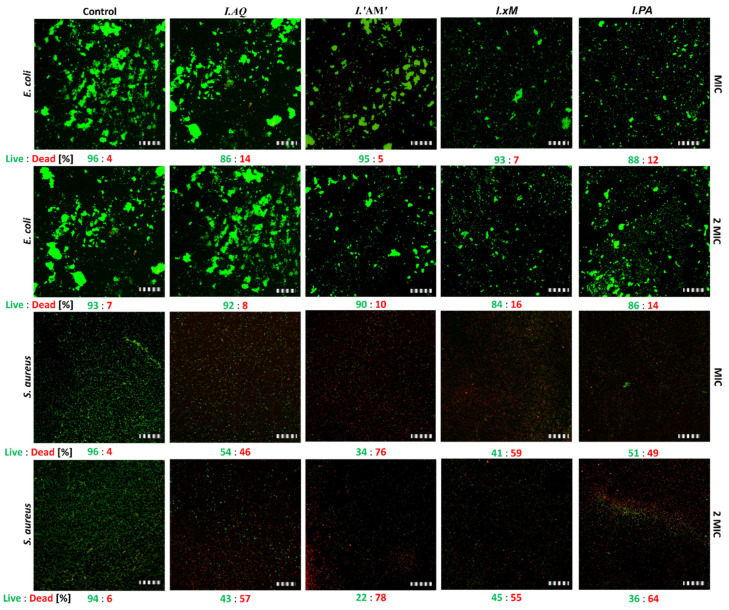
*E*. *coli* and *S*. *aureus* killing percentage in the biofilm condition analysed by counting the live/dead bacteria. Legend: *I.PA*—*I. paraguariensis*; *I.AQ*—*I. aquifolium*; *I.’AM’*—*I. aquifolium* ‘Argentea Marginata’; *I.×M*—*I.* × *meserveae* ‘Blue Angel’.

In this experiment, the *E. coli* and *S. aureus* biofilms were treated by extracts of *Ilex* spp. at two concentrations (MIC and 2 MIC). The samples were stained by Film Tracer^TM^ LIVE/DEAD^TM^ Biofilm Viability Kit. Bacteria killing percentage in biofilm condition was analysed by counting the live/dead bacteria and results are shown in Figure 6.

Comparison of bactericidal effects of *Ilex* ssp. water extracts (MIC and 2 MIC). Examples of fluorescence images showing the top view of 24 h developed biofilm formed by *E. coli* and *S. aureus*. The control is a living biofilm. The samples were stained by a FilmTracer^TM^ LIVE/DEADTM Biofilm Viability Kit. Bacteria killing percentage in biofilm conditions was analysed by counting the live/dead bacteria. Scale bar = 100 µm. Results determining the viability of the biofilm under the influence of the tested extracts showed a strong differentiation between the tested bacterial strains. The viability of the *E. coli* biofilm was similar (circa 90%) for all extracts compared to the untreated control.

## 3. Discussion

### 3.1. Comparison of Phytochemical Composition of Ilex spp.

The presence of hydroxycinnamic acids derivatives, methylxanthines, flavonoids and saponins in the *Ilex* family is widely known [5,8,17,33]. Saponins were not in the scope of this investigation because they were analysed in previous research [18], as described earlier.

The most considerable group of compounds was hydroxycinnamic acids derivatives in qualitative and quantitative terms. This was composed mainly of monoesters and diesters of hydroxycinnamic acids (usually caffeic acid) and, to a lesser degree, of glucose monohydroxycinnamic acids esters. The tested yerba mate exhibited a similar amount of hydroxycinnamic acids derivatives (81.23 mg g^−1^) to literature data (from 66 to 97 mg g^−1^ [5,8]), while European *Ilex* species contained from about a three to four times lower amount of these components. The profile of these components was also the richest for yerba mate. Among these substances, to our best knowledge, some components such as caffeoylglucoses (components 6, 15 and 18) and *p*-coumaroylquinic acids (components 16 and 27) were reported the first time for *I. aquifolium* ‘Argentea Marginata’ and *I.* × *meserveae,* as well as feruloylquinic acids (components 22, 34 and 36) for *I.* × *meserveae,*. They were previously found in another *Ilex* species, however [5,8,9,10].

In the case of flavonoids, in all tested samples, lower amounts than those of hydroxycinnamic acid derivatives were found. Most of the identified flavonoids were reported previously for *I. paraguariensis* [5,8,9]. Among all investigated samples, *I.* × *meserveae* exhibited the richest profile of flavonoids. Some derivatives of rhamnetin and isorhamnetin (components 33, 35, 38, 50, 52, 54 and 59), quercetin (components 24, 25, 40 and 55) kaempferol (components 29, 47 and 49) and myricetin (component 39) are reported for the first time in this paper. According to literature data [5,8,9,10,33,43] main flavonoids of *Ilex* species are glucosides of quercetin, kaempferol and isorhamnetin and, therefore, most new components were predicted to be derivatives of these aglycones.

Apart from polyphenol components, the main difference between yerba mate and other samples was the absence of methylxanthines in European *Ilex* species. Some older literature [10] described the presence of theobromine in *I. aquifolium*, but our research did not confirm this observation.

To summarise, European *Ilex* species contained a mix of hydroxycinnamic acids derivates and some flavonoids, similar to *I. paraguariensis*. Observed qualitative and quantitative differences were probably caused by differences in metabolism in hot and colder climates. A potentially lower amount of polyphenols and lack of methylxanthine alkaloids in temperate climates may be caused by lower pressure of pests (evolutionary alkaloids work as antipest components) [44].

### 3.2. Potential Utilisation of Ilex spp. in Obesity, Digestive Problems and Food Industry

According to some papers, yerba mate may be used to reduce body weight due to its high content of caffeoylquinic acids [45,46]. Despite a lower amount of hydroxycinnamic derivates, European species may potentially be used for this purpose due to the relatively high amount of chlorogenic acids [47], absence of methylxanthines and ease of cultivation. The main disadvantage of methylxanthines, especially caffeine, lies in their potential hypertension inducing effect, which is undesirable when reducing body mass. Moreover, a relatively high content of rutin may also be helpful in the reduction of body mass due to the potential normalisation of lipid metabolism. Rutin is known to lower liver weight and decrease enzymes activity, as well as total plasma cholesterol and LDL [48]. Chlorogenic acid is also an insulin sensitiser [46], which may also help normalise metabolism. It is also worth adding that chlorogenic acid and similar components increase the secretion and production of bile [49] and exhibit an antiulcer effect [50]. Therefore, *Ilex* spp. are potential candidates for different formulations to treat digestive system conditions (herbs or extracts mix).

The last potential application is associated with the strong antioxidant properties of mono and dicaffeoylesters of quinic acid. Due to the relatively high amount of these components in *Ilex* water extracts, they may be used in chemoprevention or as food preservatives. For example, our previous research demonstrated the possibility of using *I.* × *meserveae* as a marinade component to prevent the peroxidation of meat [51].

However, the possibility of internal use of European *Ilex* species requires considerable further and more profound research, the most critical factor being their unclear toxicological status.

### 3.3. Toxicological Status of Ilex spp.

It was reported that the main toxins of *Ilex* species are saponins [52,53] and cyanogenic glycosides [54]. Saponins from *I. opaca* are said to be hemolytic [53], although their bioavailability is very low. This is fortunate, as it substantially decreases their risk of becoming systemic toxins. In the case of *I. aquifolium*, some authors claimed that only berries are toxic due to the presence of high quantities of saponins [55] and cyanogenic glucosides [54], while the leaves are nontoxic [55]. However, other authors claimed that leaves are also toxic [52]. It is known that *I. aquifolium* leaves upset the gastrointestinal system (vomiting, diarrhoea, depression, and anorexia) in animals after treatment [52]. Authors connected this effect with irritation of the gastrointestinal system by the rigid texture of leaves and the presence of saponins [52]. However, the potentially toxic effects of *I. aquifolium* leaves extracts remain unknown. In our opinion, it is possible that the harmful effect may be exhibited only by some chemotypes of *I. aquifolium,* which contain a significant amount of saponins and/or cyanogenic glycosides, or specific profile of these components. It is widely known that holly leaves were successfully used as fodder in some parts of the UK, which supports our point [56]. Moreover, saponins are also present in yerba mate, which is not known for toxic effects even in the case of prolonged periods of administration [3,57]. In traditional Chinese medicine, applications of *Ilex* species include gastrointestinal disorders [57]. The toxicological status of *I.* × *meserveae* and *I. paraguariensis* is clearer than that of *I. aquifolium*. It is known that saponins present in the hydroalcoholic dry extract as well as in water infusions from leaves of these species do not demonstrate nephrotoxicity but, conversely, have a protective role on kidney status in animals fed a regular diet and in a high-cholesterol diet [18].

In summary, *I.* × *meserveae* and *I. paraguariensis* are promising candidates for internal application, while *I. aquifolium* and *I. aquifolium* ‘Argentea Marginata’ additional tests are required.

### 3.4. Antimicrobial Potential of Ilex spp.

It is quite significant that the strongest antibacterial agents (in terms of MIC and biofilm tests) are usually *I. aquifolium* and *I. aquifolium* ‘Argentea Marginata’. Comparison with the literature has shown that our water extracts of yerba mate had a higher MIC value against *S. aureus* than water extracts investigated by others [13,14]. In the case of *E*. *coli,* the results were inconclusive; MIC was higher [13] or lower [14]. Different extraction techniques probably caused the observed effect. Moreover, all extracts exhibited lower activity against *E. coli* and fungi (in most cases) than *S. aureus*, as well as a shorter (8–16 h) inhibited growth period of *E. coli* and fungi. This proved the higher resistance of *E. coli* and fungi, which may be caused by a non-specific efflux pump in *E. coli* and differences in the structure of cell barriers between Gram-positive and Gram-negative bacteria and fungi.

Correlation between the composition of *Ilex* spp. water extracts and antimicrobial activity was ambiguous. It is known that hydroxycinnamic acid derivatives such as chlorogenic acid possess antibacterial and antibiofilm activity [58,59]; the antimicrobial potential of flavonoids is also well documented [60]. However, in our tests, *I. paraguariensis* did not show the highest activity in MIC and biofilm assays despite the fact it contained the greatest quantity of polyphenols. This seems to indicate that antimicrobial activity is probably correlated with a different group of components. Apart from polyphenols, all tested *Ilex* species contain microbiologically active saponins as well, which may be responsible for their properties. Saponins possess a documented ability to decrease water surface tension and disrupt the biological functions of cell membranes. Therefore, it is expected that saponins should extirpate bacterial biofilms, which was proven for different saponins such as those from *Quillaja* species [61]. Moreover, the antibiofilm effect is also known for extracts from *I. guayusa* with saponins suspected to be active components [17] as well as antifungal effects of saponins aglycones from *I. integra* [15]. Based on the fact that *I. aquifolium* and *I. aquifolium* ‘Argentea Marginata’ contained the greatest quantities of saponins among the investigated subspecies as published prior to this work [18], we suspect that these components may be responsible for the observed strong antibacterial properties of both species. It may also be the case that hydroxycinnamic acid derivatives and flavonoids may work as auxiliary antibacterial agents. The literature [62] discusses synergistic antimicrobial effects of saponins and polyphenols, and the conclusions support our reasoning. The mechanism of this synergistic effect is not fully known, but we suspect it has a multifactorial nature. From one point of view, saponins may increase the concentration of hydroxycinnamic acid derivatives and flavonoids in bacterial cells by disrupting cell membranes and biofilms. On the other hand, saponins may also increase polyphenol activity against cell barriers via an unknown mechanism or mechanisms.

The ability of biofilm formation is one of the most important virulence factors of human pathogenic bacteria. Moreover, it is also responsible for the easy spread of bacteria across hospital and non-hospital environments. For these reasons, antibiofilm tests should be separately discussed.

Investigation of the effect of extracts on adhesion and biofilm formation under flow conditions showed significant inhibition of the adhesion process caused by *I. aquifolium* and *I. aquifolium* ‘Argentea Marginata’ extracts for both strains. Reduction of bacterial surface adhesion caused by *Ilex* spp. extracts may be a result of different factors. In the beginning, polyphenols and saponins may directly inactivate adhesins or inhibit their production in the case of *S. aureus*, as well as disrupt the function of fimbriae of *E. coli* [63]. Additionally, they may also disrupt the quorum quenching mechanism [64].

Biofilm formation assays under flow conditions did not allow us to investigate all aspects of biofilm formation, and therefore we decided to use additional tests. We chose to use polystyrene surface tests to evaluate the production of biofilms in static conditions during 24h.

Polystyrene tests are a standard in *E. coli* biofilm research, and this material is widely used in the medicinal industry for the production of covers, diagnostic instruments, and disposable tableware, among others. This aspect is also important for *S. aureus*, which is known for the ease of spreading in hospital environments and often cause serious infections of wounds. Assays performed on polystyrene surfaces showed that all extracts except that from *I. paraguariensis* caused a decline in biofilm production. It is also worth noting that, in all cases, the effect was stronger against *E. coli* than *S. aureus*.

Eradication and viability were the last investigated aspects of antibiofilm assays. It is known that, usually, eradication of a biofilm is more difficult than prevention of its formation due to the protection of bacterial cells by extracellular polymeric substances (EPS) and other rather complex phenomena [65]. All the extracts that we researched eradicated the biofilm of *E. coli* to a lesser degree than that of *S. aureus*. Moreover, *E. coli* biofilms were found to exhibit lower viability on *Ilex* extracts than those of *S. aureus*. This proves that mature biofilms of *S. aureus* are more sensitive to *Ilex* extracts than those of *E. coli.* This is probably caused by compounds from the extracts such as saponins and polyphenols penetrating through water channels inside the biofilm structure, reducing its viability [64]. Emulsifying properties of saponins may also facilitate this process. Higher resistance of *E. coli* biofilms is probably a result of different compositions of the biofilm matrix.

In summary, due to the unclear toxicity status of *I. aquifolium* and *I. aquifolium* ‘Argentea Marginata’, their antimicrobial potential should be used for purposes of external application only, at least until there are enough data to assure safety of the more systemic application. One f such function may be the production of multicomponent lavaseptics or surface antiseptics due to the high ability of the extracts of reducing *S. aureus* biofilm viability. Another external application safe for humans is using the *Ilex* species water extracts in antiseptics and skin cosmetics. Only when the toxicological status of *I. aquifolium* and *I. aquifolium* ‘Argentea Marginata’ water extracts is fully explained and classified as safe for humans should internal application be considered. Due to activity against *E. coli* and diuretic effects [11,66], European *Ilex* species may be a candidate for medicines in diarrhoea and/or urinary tract infections. In Chinese medicine, some *Ilex* species (*I. latifolia* and *I. kudingcha*) are traditionally used in diarrhoea [57] and yerba mate beverages are used in urinary infections [4] and exhibit a diuretic effect [11]. However, European *Ilex* species possess a distinct advantage, as their lack of caffeine prevents them from increasing symptoms of diarrhoea, which caffeine is known to cause [67]. *I.* × *meserveae* due to relatively high amount of chlorogenic acid and rutin, as well as more clear toxicological status internal application, especially in the reduction of body mass and as an appetiser, should be considered.

## 4. Materials and Methods

### 4.1. Plant Material and Reagents

Leaves of *Ilex aquifolium* L., *I. aquifolium* ‘Argentea Marginata’ and *I.* × *meserveae* ‘Blue Angel’ (*I. aquifolium* × *I. rugosa*) were obtained from the plant collection of the Department of Horticulture, Wrocław University of Environmental and Life Sciences. Species and cultivar identification was performed by prof. Przemysław Bąbelewski from the Department of Horticulture, Wrocław University of Environmental and Life Sciences. Plants in the *Ilex* collection were marked according to the latest tree and shrub catalogues compiled by Polish scientists working for the Polish Nurserymen’s Association. Fresh plant material was air-dried at 35–40 °C for 24 h. *I. paraguariensis* ‘la oracion’ (dry plant material) was purchased from Carbales Company (Buenos Aires, Argentina).

Acetonitrile HPLC grade was obtained from Sigma-Aldrich (Saint Louis, MO, USA) and MS grade chemicals (Acetonitrile, 99% formic acid, methanol, water) were purchased from Merck (Darmstadt, Germany). UHPLC deionised water was prepared on-site.

Analytical standards included caffeine and theobromine (Sigma-Aldrich, St. Louis, MO, USA), caffeic acid (Fluka, St. Gallen, Switzerland), chlorogenic acid (Roth, Karlsruhe, Germany), rutin (Merck, Darmstadt, Germany), *p*-coumaric acid (Extrasynthese, Genay, France).

Mueller-Hinton agar and Sabouraud agar were obtained from Oxoid (Hampshire, UK).

### 4.2. Preparation of Water Extracts

Boiling water was poured onto the plant material (20 mL per 1 g), then kept at 80 °C for 3 h with mixing. After this time, extracts were stored at room temperature for 21 h and then filtered by Whatmann filtrate paper. One mL was used for UHPLC-DAD-MS/MS analysis; this was frozen at −20 °C and lyophilised for 24 h. After lyophilisation, extracts were mechanically standardised by grinding in a mortar. Lyophilised plant extracts were then used for antimicrobial assays.

### 4.3. Qualitative UHPLC-DAD-MS/MS Analysis of Water Extracts

Analysis was performed on Thermo Scientific™ UltiMate™ 3000 system (Thermo Fischer Scientific™ Dionex™, Waltham, MA, USA), equipped with an autosampler and DAD detector set at 280, 320 and 360 nm. Spectral data were recorded in the 200–600 nm range. Chromatographic separation was performed on Accucore RP-MS 2.6 μm, 100 × 2.1 mm, column (Thermo Fisher Scientific, Waltham, MA, USA) thermostated at 35 ± 2 °C. Injection volume was set to 1 μL. The mobile phase consisted of 0.1% formic acid in water (solvent A) and acetonitrile (solvent B). The flow rate was set at 0.4 mL.min^−1^ and the separation was obtained using gradient: 100% of solvent A, decreasing to 70% within 22 min, decreasing to 0% at minute 25.0, isocratic for 2 min and increasing to 100% at minute 33.0.

UHPLC-DAD-MS/MS was performed at a similar setting and chromatographic conditions, additionally using a Compact QqTOF MS/MS detector (Bruker, Darmstadt, Germany). The MS detector was used in electrospray negative mode. Parameters of analysis for both modes were the same: the ion source temperature was set at 210 °C, nebuliser gas pressure was set at 2.0 bar, dry gas (nitrogen) flow 8.0 L/min and temperature at 210 °C. The capillary voltage was set at 4.5 kV. The collision energy was set at 8.0 eV. Internal calibration was obtained using 10 mM solution of sodium formate. For ESI-MS/MS experiments, collision energy was set at 35.0 eV and nitrogen was used as the collision gas. The scan range was set from 30 to 1300 *m*/*z*. Before the analysis, all the extracts were dissolved in ethanol and filtered through CHROMAFIL^®^ 0.22 µm, Ø13 mm, H-PTFE membrane syringe filter (Macherey-Nagel, Düren, Germany).

### 4.4. Quantification of Polyphenols in Ilex Species

Quantification was performed according to the methods described in Section 4.3. Standard compounds were dissolved in pure methanol and subsequently diluted to obtain calibration curves in the range of concentrations from 1.000 to 0.016 µg mL^−1^. Amounts of different compounds in the samples were calculated based on the calibration curve of the appropriate standard or corresponding parent compound. Caffeoyl and feruloyl quinic acids and caffeoylglucoses were expressed as chlorogenic acid equivalents, monomers of *p*-coumaroyl quinic acid as *p*-coumaric acid equivalent and flavonoids as rutin equivalents.

### 4.5. Strains and Growth Conditions

In this study, we used the following bacterial (*Escherichia coli* ATCC 10536, *Staphylococcus aureus* DSM799) and fungal strains (*Candida albicans* DSM1386, *Alternaria alternata* CBS1526, *Fusarium oxysporum* KB-F1, *Aspergillus niger* DSM1957). Bacterial strains were cultured in Mueller Hinton II Broth BD (MHB) and fungal strains in MHB enriched with 2% glucose. The strains were incubated aerobically for 24 h at 37 °C (*E. coli*, *S. aureus* and *C. albicans*) or for 48 h at 28 °C for the remaining ones. Overnight, microorganism cultures were centrifuged, washed with PBS (pH 7.4) and suspended in fresh MHB to obtain suitable optical density.

### 4.6. Minimal Inhibitory and Fungicidal Concentrations

Values of the minimal inhibitory concentrations (MIC) were determined according to the modified protocol described previously [68,69].

Overnight, bacterial strains cultures were suspended in PBS (pH 7.4) (0.5 McF) and for fungal turbidity adjusted to an OD (Optical Density) of 0.8, all finally diluted to obtain 10^5^ CFU mL^−1^. Microorganism suspensions (10 µL) were added into the wells of the microplate containing serial dilutions (2556–2564 µg mL ^−1^) of the tested water extracts and filled with 100 µL of MHB medium. Microplates were incubated aerobically for 24 h at 37 °C (*E. coli*, *S. aureus*, and *C. albicans*) or, for other strains, 48 h at 28 °C. As a growth control, a fungal suspension in MHB without extracts was used; for the sterile control, MHB without microorganisms, and for the blank control, a fungal suspension in PBS. Each control and extract dilution were performed in duplicate and the experiment was repeated three times. The MIC values were determined spectrophotometrically (590 nm) using Asys Hitachi 340, Driver version: 4.02 (Biogenet, Poland). To determine the minimal bactericidal and fungicidal concentration (MBC and MFC), 100 µL of fungal suspension from each dilution of the tested compound was transferred to a Mueller Hinton II Agar BD (MHA) plate. MBC and MFC were expressed as the concentration of the *Ilex* spp. extracts that reduced the number of colony-forming units by 99.9% after 48 h of incubation at 37 °C or at 28 °C.

### 4.7. Evaluation of Bacterial and Fungal Growth Curves

The biological activity of each tested extract of *Ilex* sp. was assessed on selected bacterial and fungal strains. The cultures were grown in an MHB medium. The tests were performed in an automated Bioscreen C system (Automated Growth Curve Analysis System, Lab systems, Finland). The working volume in the wells of the Bioscreen plate was 300 µL, comprised of 280 µL of culture medium and 10 µL of cell solution (to obtain final concentration 1 × 10^5^ CFU mL^−1^). Extracts were dissolved in water and used at a final concentration of 0.1% (*w*/*v*). The temperature was controlled at 37 °C for bacteria and 28 °C for fungi. The optical density of the cell suspensions was measured automatically at λ = 560 nm in regular intervals of 30 min for the dependence on optical density of cultivation time. The cell cultures were continuously shaken. Each culture was grown in five replications. The data were analysed using spreadsheet software (Excel 2013 MS Office) and the averages of the triplicates for each type of culture medium were calculated. The averages were used to generate the growth curves for each strain studied, constructed as a function of the incubation time and the absorbance of the culture medium. The resulting microbial growth curves were compared to control cultures in a medium supplemented with water (without extract) [70].

### 4.8. Influence of Extracts on Adhesion Process and Biofilm Formation of Bacterial Strains in the Microfluidic Flow System

A microfluidic flow system BioFlux 1000, with 48-well plates (Fluxion Biosciences, Alamenda, AZ, USA), was used to study adhesion and biofilm formation in continuous flow. The bacterial strains (*E. coli* and *S. aureus*) were diluted to obtain 10^5^ CFU mL ^−1^ in 1 mL of Tryptic Soy Broth BD™ medium (TSB) and flushed into the channels. The extracts were applied at half of their MICs to avoid growth inhibition. Initially, the inoculum (900 µL) was incubated without flow for adhesion of bacteria, the wells were filled, and flow initiated at 0.5 dyn per 1 cm^2^. The plate was incubated at 37 °C for 17 h period in the incubator chamber (Carl Zeiss Pecon Incubator XL S1, Erbach, Germany). Parameters such as flow shear, adhesion time, and inoculum concentration were tested for biofilm production in the microfluidic system. Imaging was performed at 1 h intervals on an Axio Inverted Observer fluorescence microscope 7 (Carl Zeiss, Erbach, Germany) equipped with an Orca Flash 40 camera (Hamamatsu, Hamamatsu-city, Japan) and 20× objective (Differential Interference Contrast (DIC) mode). Scale bar = 100 µm. Three positions per channel were imaged per extract. Quantification of total biofilm was performed with the BioFlux software (Fluxion, Alameda, AZ USA) [71].

### 4.9. Influence of Extracts on Biofilm Formation on Polystyrene

To evaluate the influence of *Ilex* spp. water extracts on *E. coli* and *S. aureus* ability to produce biofilm, the strains (10^5^ CFU mL^−1^) were incubated for 24 h in the presence of extracts in the TSB medium at 37 °C. The extracts were applied at half of their MICs to avoid growth inhibition. Samples (200 uL) were transferred to a 96-well microplate and incubated with shaking (400 RPM) for 24 h at 37 °C. Microplates were washed three times with distilled water and each well was stained with crystal violet for 10 min. Wells were washed three times and the bound crystal violet was dissolved with a 100% 2-propanol, 1% SDS, 50 mM HCl mixture. Absorbance was measured at λ = 590 nm as described by Piecuch et al. Untreated bacterial cells were used as the biofilm growth control. The experiment was conducted with three independent repetitions [72].

### 4.10. Influence of Extracts on Biofilm Eradication

Bacterial biofilm eradication on polystyrene surfaces (96-hole microplate) was assessed with 200 µL of *E. coli* and *S. aureus* cultures, diluted to obtain 10^5^ CFU mL ^−1^ in TSB medium, and pipetted into wells of microplates. Cultures were incubated at 37 °C for 24 h with shaking (400 rpm). After this time, microplates were washed three times with sterile physiological salt solution, then the *Ilex* spp. extracts were added to final concentrations MIC and 2 MIC (respectively for strains) and incubated for 2h at 37 °C with shaking (400 rpm). The microplates’ holes were washed three times with sterile physiologic salt solution and then the biofilm was stained with a 10 min. incubation with 100 μL 2.45 μM of crystal violet. The microplates were then gently washed three times with sterile physiological salt solution. Crystal violet bound with biofilm on the surface of the plate was dissolved by washing with 200 µL of washing solution (isopropanol, HCl 50 mM, SDS 1%). Absorbance was then read at λ = 590 nm (ASYS UVM 340 Biogenet). Untreated cells were used as control. The experiment was carried out in triplicate [73].

### 4.11. Biofilm Viability on Fluorescence Microscopy after the Action of Ilex Extracts

Aliquots of 3 mL of each cultured *E. coli* and *S. aureus* in TSB medium 10^5^ CFU/mL were added to the wells of sterile six-well plates. Sterile microscopic slides (Ø 15 mm) were put in the wells and the resultant cultures were incubated at 37 °C for 24 h with shaking (400 RPM). The slides were washed twice with sterile physiological salt solution and transferred to fresh six-well plates. The biofilm on glass for each culture was treated with *Ilex* spp. extracts in two concentrations (MIC and 2 MIC), then were stained with 3 μL of propidium iodide (Ex λ = 543 nm) and 3 μL SYTO 9 (Ex λ = 488) for 3 min using LIVE/DEAD BacLight Bacterial Viability Kit (Thermo Fisher Scientific). Imaging was performed on an Axio Inverted Observer fluorescence microscope 7 (Carl Zeiss, Erbach, Germany) equipped with an Orca Flash 40 camera (Hamamatsu, Hamamatsu-city, Japan) and 10× objective Scale bar = 100 µm. Acquired images were processed and analysed using Fiji/ImageJ software ver. 1.53c (NIH). First, maximum intensity projections (MIP) were obtained from stacks of images. The areas from binarised images were then transferred onto original live and dead channel MIP images and mean fluorescence intensities of all detected objects per field of view were calculated using the ImageJ’s Analyze Particles function [73].

## 5. Statistical Analysis

Variance analysis was performed using software Statistica 13 ver. 13.3.721.0 (ANOVA one-way analysis). A probability value of *p* < 0.05 was considered significant. One-way ANOVA analysis was performed for influence of extracts on biofilm formation and the influence of extracts on biofilm eradication. Standard deviations were calculated for qualitative and quantitative UHPLC-DAD-MS/MS analyses.

## 6. Conclusions

Our research established that water extracts obtained from leaves of *Ilex aquifolium* L., *I. aquifolium* ‘Argentea Marginata’, *I.* × *meserveae* ‘Blue Angel’ are a reliably rich source of polyphenols, especially chlorogenic acids and their derivates. Their potential applications should, therefore, be associated with known activities of these groups of components, including externally and internally applied preparations. Due to the presence of saponins, external applications at this moment are to be considered first, for those are safer. Taking obtained data under consideration, we can assume that extracts from *Ilex* genus can play an important role as a potential source of antimicrobial agents. Potentially, a purified fraction of *Ilex* may be used in multicomponent lavaseptics, antiseptics and cosmetics. Internal application of *Ilex aquifolium* L., *I. aquifolium* ‘Argentea Marginata’, *I.* × *meserveae* ‘Blue Angel’ requires further research due to their unclear toxicological status for humans. Potentially, those could still be used as components of digestive composition (both herbs and extracts) as well as for auxiliary treatments in urinary tract infections caused by *E*. *coli*.

In the future, we wish to develop studies of the antimicrobial effects of *Ilex* spp. The planned research includes multiple approaches. The basic path is the evaluation of antimicrobial effects against other microorganisms, as well as comparison of different types of extracts and screening of other *Ilex* species, their cultivars and components such as fruits. Another approach is the purification of water extracts from ballast material such as sugars and potential division into fractions and subfractions. Next, the obtained fractions and subtractions may be used to research interactions between saponins and polyphenols in their antimicrobial activity mechanism of action. Moreover, research of potential synergism between *Ilex* extracts and other antimicrobial agents (such as antibiotics) should also be performed. The rising resistance of microorganisms to antibiotics has required searching for new therapies and new antibacterial agents. Multicomponent mixtures of antibacterial agents may comprise these therapies due to the expected weaker production of multi-drug resistant strains and potential lower doses of single antibacterial agents.

## Figures and Tables

**Table 1:** UHPLC-DAD-MS/MS identification of components in *Ilex* leaves water extracts.

NB	Component	RT	UV λ_nm_ Max	MS ^1^ *m/z*	MS ^2^ Base Peak *m*/*z*	MS2 Secondary Peaks—*m/z* (Abundance)	Ref.
1	Quinic acid ^a,b,c^	0.92	258	191.0207	111.0087	-	standard
2	Isomer of lithospermoside I ^b,c^	2.58	258	328.1101	130.0336	121.034 (20.16), 148.043 (19.10)	[19]
3	Isomer of lithospermoside II ^b,c^	2.75	259	328.1043	130.0305	121.0295 (40.06), 102.0358 (36.89), 125.0218 (3.17)	[19]
4	Unidentified	3.39	258	371.0984	191.0563	135.0455 (5.18), 161.0237 (3.29), 173.0456 (2.60)	-
5	* 3-Hydroxy-4-*O*-glucosylbenzoic acid ^b,c^	3.90	259	315.0720	108.0227	152.0121 (80.47)	[20,21,22]
6	Caffeoylglucose I ^b,c^	4.09	326	341.0889	161.0264	135.0467 (22.08), 179.0353 (13.14)	[8,22,23]
7	* Canthoside B ^b,c^	4.75	257	463.1454	233.0671	293.0870 (58.98), 125.0252 (50.23), 123.0448 (34.74), 149.0461 (32.35), 191.0550 (17.67), 131.0351 (10.15), 105.0348	[24,25]
8	Caffeoylglucose II ^b,c^	4.81	327	341.0885	161.0266	135.0475 (55.38), 179.0356 (14.39), 221.051 (7.01)	[8,22,23]
9	Theobromine ^a^	5.07	273	-	-	-	standard
10	Caffeoylglucose III ^b,c^	5.14	327	341.0878	161.0267	135.0473 (49.92), 179.0386 (18.17), 221.051 (3.04)	[8,22,23]
11	* *p*-Hydroxybenzoic acid ^a,b,c^	5.50	280	137.0246	108.0238	136.0166 (75.91)	[26]
12	*cis*-3-Caffeoylquinic acid ^b,c^	5.51	321	353.0886	191.0594	135.0474 (63.62), 179.0375 (38.53)	[8]
13	Neochlorogenic acid (5-CQA) ^b,c^	5.94	324	353.0888	191.0586	135.0477 (69.82), 179.0377 (45.58), 161.0266 (5.09)	[8]
14	Caffeoylglucose IV ^b,c^	6.47	326	341.0890	161.0266	135.0472 (24.92), 179.0381 (17.14), 203.0384 (3.96), 221.0474 (2.19)	[8,22,23]
15	6-Caffeoylglucose ^b,c^	7.21	324	341.0890	161.0263	135.0481 (63.45), 179.0377 (63.45), 221.0475 (42.31), 281.0661 (5.44)	[8,22,23]
16	3-*p*-Coumaroylquinic acid ^b,c^	7.46	313	337.0938	119.0519	163.0417 (82.87), 191.0586 (14.73)	[8]
17	Caffeoylquinic acid ^b,c^	7.61	324	353.0882	135.0464	191.0570 (96.13), 173.0450 (80.28), 179.0354 (67.36), 93.0365 (10.89), 155.0352 (8.34), 111.0467 (8.22), 137.0241 (7.54)	[8]
18	Caffeoylglucose VI ^b,c^	8.05	324	341.0890	161.0268	135.0475 (80.13), 179.0367 (76.09) 221.0472 (44.08), 281.0662 (8.54), 203.0385 (2.33)	[8,22,23]
19	Caffeic acid ^a, b, c^	8.17	321	179.0371	135.0473	117.0362 (6.09)	standard
20	Chlorogenic acid (3-CQA) ^a,b,c^	8.30	324	353.0883	191.0584	161.0265 (2.08)	standard
21	Caffeine ^a^	8.52	278	-	-	-	standard
22	3-Feruloylquinic acid ^b,c^	8.63	325	367.1036	134.0399	193.0528 (72.78), 149.0625 (10.02), 117.0361 (9.64)	[8]
23	Cryptochlorogenic acid (4-CQA) ^b,c^	8.71	324	353.0892	135.0473	191.0583 (97.91), 179.0374 (81.84), 161.0269 (8.46),	[8,9]
24	Quercetin-di-glucoside ^b,c^	9.62	352, 253	625.1411	625.1416	463.0865 (80.18), 301.0368 (15.78), 299.0190 (9.67)	[27,28]
25	Quercetin-deoxyhexoside-hexoside ^b,c^	9.81	353, 255	609.1471	-	-	[29]
26	*cis*-5-Caffeoylquinic acid ^b,c^	9.97	325	353.0878	191.0585	-	[8]
27	4-*p*-Coumaroylquinic acid ^b,c^	10.23	312	337.0940	173.0478	191.058 (55.34), 119.0522 (37.71), 163.0418 (34.04), 137.0269 (13.06)	[8]
28	* Flavonoid-derivate ^b,c^	10.51	353, 255	667.1523	-	-	[29]
29	Kaempferol-di-hexoside ^b,c^	10.66	-	609.1463	447.0917	285.0413 (73.38), 609.1441 (41.45), 283.0255 (24.85), 327.0522 (8.38), 489.0952 (4.21)	[28]
30	Kaempferol-rutinoside-hexoside ^b,c^	10.86	346, 265	755.2039	593.1502	755.2028 (25.24), 447.0918 (4.80), 285.0397 (4.05)	[30,31]
31	Unidentified	10.92	282	537.1980	375.1456	357.1344 (33.02), 537.1990 (32.01), 489.2040 (25.25), 119.0356 (21.60), 327.1211 (19.19), 321.1131 (17.17), 339.1247	-
32	Unidentified	11.06	-	593.1519	593.1487	473.1092 (30.56), 353.0676 (16.77), 383.0765 (7.87), 503.1181	-
33	* Isorhamnetin-3-*O*-gentiobioside ^b,c^	11.06	345, 254	639.1576	477.1040	315.0504 (61.04), 639.1571 (28.77), 313.0387 (12.37)	[32,33]
34	4-Feruloylquinic acid ^b,c^	11.20	324	367.1045	173.0476	134.0394 (41.39), 193.0527 (22.24)	[8,9]
35	* Isorhamnetin-rutinoside-glucoside ^b,c^	11.21	352, 254	785.2152	623.1603	785.2078 (15.49), 315.0503 (6.79), 477.0992 (6.61)	[32,33]
36	5-Feruloylquinic acid ^b,c^	11.31	325	367.1046	191.0583	134.0403 (24.8), 193.0538 (23.18),173.0476 (16.98), 149.0632 (4.52)	[8,9]
37	Caffeoylquinic lactone ^b,c^	11.41	-	335.0787	-	-	[8]
38	Isorhamnetin derivate ^b,c^	12.00	349, 253	725.1572	519.1137	681.1659 (63.20) 477.1007 (27.15) 315.0503 (16.33), 561.1252 (13.86), 357.0587 (5.06)	-
39	Myricetin-hexoside ^b,c^	12.38	355, 254	479.1206	317.0683	299.0591 (15.88), 479.1203 (13.93), 289.0719 (11.26) 0.001640, 165.0210	[34]
40	Quercetin-pentoside-hexoside ^b,c^	12.84	352, 256	595.1310	300.0282	595.1303 (55.26), 179.0017 (2.11)	[35,36]
41	Quercetin-7-*O*-rutinoside ^b,c^	13.34	353, 256	609.1457	609.1456	300.0291 (47.5)	[29]
42	Rutin (Quercetin-3-*O*-rutinoside) ^a,b,c^	13.67	353, 256	609.1445	609.1441	300.0312 (43.16)	standard
43	Quercetin-glucoside ^b,c^	13.95	352, 256	463.0878	300.0301	-	[9,29]
44	Unidentified	14.51	392, 263	701.1363	701.1355	335.0193 (67.64), 509.0718 (9.63), 191.0564 (4.69), 669.0322 (2.02)	-
45	Unidentified	14.51	385, 251	493.1338	331.0837	-	-
46	Flavonoid ^c^	14.80	354, 257	547.2386	149.0481	191.0585 (30.48), 131.0365 (15.69), 101.0264 (11.61)	-
47	Kaempferol-3-*O*-rutinoside ^b,c^	15.05	348, 265	593.1495	285.0415	-	[8,9,37]
48	3,4-Dicaffeoylquinic acid ^b,c^	15.19	325	515.1185	173.0477	179.0372 (99.12), 353.0887 (54.25), 191.0583 (46.01), 161.0262 (19.59), 135.0474 (16.21)	[8,9]
49	Kaempferol-3-*O*-glucoside ^b,c^	15.38	345, 254	447.0928	284.0331	447.0938 (14.96), 255.0305 (12.35), 227.0358 (6.66)	[8,38]
50	Isorhamnetin-3-*O*-rutinoside ^b,c^	15.38	353	623.1621	315.0518	623.1618 (90.32)	[39]
51	3,5-Dicaffeoylquinic acid ^b,c^	15.46	326	515.1184	191.058	179.0371 (66.61), 353.0882 (45.97), 135.0472 (11.41), 161.0263 (4.97)	[8,9]
52	Isorhamnetin-3-*O*-glucoside ^b,c^	15.76	353, 255	477.1032	314.0442	477.1005 (36.22), 286.0504 (8.18), 299.0195 (4.73)	[33,40]
53	Dicaffeoylquinic acid ^b^	15.76	325	515.1204	191.0566	179.0358 (73.65), 353.0878 (30.52), 173.0459 (16.28), 135.0447 (11.64), 161.0257 (11.13), 335.0779 (5.08), 359.0566 (2.56), 155.0362 (2.03)	[8]
54	Isorhamnetin glucoside ^b,c^	16.10	253	477.1051	314.0431	299.0193 (93.25), 477.1052 (51.68), 271.0265 (10.28)	[40,41]
55	Quercetin-rutinoside ^b,c^	16.25	284	609.1829	301.0717	609.1807 (5.26)	[29]
56	Dicaffeoylquinic acid ^b,c^	16.31	325	515.1197	173.0458	179.0358 (84.27), 707.1842 (54.47), 191.0560 (38.01) 161.0247 (13.78), 135.0454 (10.60), 354.0899 (8.68), 155.0340 (4.89), 335.0758 (2.88), 163.0756 (2.37), 243.0645 (2.37)	[8]
57	4,5-dicaffeoylquinic acid ^b,c^	16.66	326	515.1188	173.0479	179.0373 (77.99), 353.0885 (65.03), 191.0586 (30.17), 135.0477 (10.58), 161.0271 (2.79)	[8,9]
58	* Hexoside derivate ^c^	16.95	-	431.1917	207.1381	251.1300 (94.96), 113.0225 (16.89), 335.0227 (15.87), 119.0341, 331 (11.20), 99.0457 (10.80), 101.0234 (9.81), 163.1470 (2.64)	-
59	Isorhamnetin 3-*O*-acetylglucoside	17.05	351	519.1147	314.0451	299.0217 (15.41), 519.1172 (6.32)	[41,42]
60	* Hexoside derivate ^c^	17.13	310	429.1771	205.1236	249.1115 (74.12), 161.1358 (44.20), 99.0439 (9.41), 153.0933 (3.39)	-
61	* Hexoside derivate ^c^	17.49	-	431.1919	207.1392	251.1292 (81.26), 99.0452 (15.10), 113.0234 (10.61), 189.1290 (9.72), 119.0352 (9.70), 101.0266	-
62	Caffeoylferuoyl-quinic acid ^b^	17.79	326	529.1342	193.0529	367.1029 (17.65), 173.0467 (6.66), 134.0391 (3.39), 191.0622 (2.19)	[8]
63	Caffeoylferuoyl-quinic acid ^b^	17.98	326	529.1322	191.0583	179.0366 (44.92), 353.0898 (14.56), 173.0496 (12.08), 193.0512 (11.45), 135.0465 (10.73), 367.1057 (10.36) 161.0259 (8.01)	[8]
64	Caffeoylquinic acid ^b,c^	18.51	326	515.1176	173.0479	179.0374 (90.57), 353.0892 (58.97), 191.0573 (45.39), 135.0479 (13.9), 161.0254 (13.9 5.49)	[8]
65	Caffeoylferuoyl-quinic acid ^b^	18.71	325	529.1338	173.0478	193.0519 (21.1), 367.1043 (19.77), 137.0254 (2.43)	[8]
66	Caffeoylferuoyl-quinic acid ^b^	19.01	-	529.1322	173.0475	179.0368 (79.05), 191.057 (73.88), 353.0882 (52.19), 135.0483 (10.6), 367.103 (9.32), 161.0303 (6.47) 137.0279 (4.76)	[8]

Table legend; NB—number; RT—retention time; * tentatively identified component (lack of accurate spectral literature database; suspected isomer of a known component or unclear UV spectrum); ^a^ component identified by comparison with a standard; ^b^ component identified by comparison of UV and/or MS/MS spectra with literature; ^c^ component identified by prediction with MS/MS spectra;—lack of experimental UV spectra/ions or not applicable.

**Table 2:** Presence of components in *Ilex* leaves water extracts.

Nb	Component	RT	*I.AQ*	*I.*’*AM*’	*I.*×*M*	*I.PA*
1	Quinic acid	0.92	+	+	+	+
2	Isomer of lithospermoside I	2.58	+	+	tr	-
3	Isomer of lithospermoside II	2.75	+	+	+	-
4	Unidentified	3.39	+	+	+	tr
5	* 3-Hydroxy-4-*O*-glucosylbenzoic acid	3.90	+	+	+	tr
6	Caffeoylglucose I	4.09	tr	tr	tr	+
7	* Canthoside B	4.75	+	+	+	tr
8	Caffeoylglucose II	4.81	-	-	-	+
9	Theobromine	5.07	-	-	-	+
10	Caffeoylglucose III	5.14	-	-	-	+
11	*p*-Hydroxybenzoic acid	5.50	+	tr	+	+
12	*cis*-3-Caffeoylquinic acid	5.51	tr	tr	tr	+
13	Neochlorogenic acid (5-CQA)	5.94	+	+	+	+
14	Caffeoylglucose IV	6.47	tr	-	-	+
15	6-Caffeoylglucose	7.21	tr	tr	-	+
16	3-*p*-Coumaroylquinic acid	7.46	+	+	+	+
17	Caffeoylquinic acid isomer I	7.61	tr	tr	tr	+
18	Caffeoylglucose VI	8.05	tr	tr	tr	+
19	Caffeic acid	8.17	tr	tr	tr	+
20	Chlorogenic acid (3-CQA)	8.30	+	+	+	+
21	Caffeine	8.52	-	-	-	+
22	3-Feruloylquinic acid	8.63	+	+	tr	+
23	Cryptochlorogenic acid (4-CQA)	8.71	+	+	+	+
24	Quercetin-di-glucoside	9.62	tr	-	+	-
25	Quercetin-deoxyhexoside-hexoside	9.81	tr	tr	+	tr
26	*cis*-5-Caffeoylquinic acid	9.97	+	+	+	+
27	4-*p*-Coumaroylquinic acid	10.23	+	+	+	+
28	Flavonoid-derivate	10.51	-	-	+	-
29	Kaempferol-di-hexoside	10.66	-	-	+	-
30	Kaempferol-rutinoside-hexoside	10.86	-	-	+	tr
31	Unidentified	10.92	+	+	+	-
32	Unidentified	11.06	-	-	+	-
33	* Isorhamnetin-3-O-gentiobioside	11.06	-	-	+	-
34	4-Feruloylquinic acid	11.20	+	+	tr	+
35	* Isorhamnetin-rutinoside-glucoside	11.21	-	-	+	-
36	5-Feruloylquinic acid	11.31	+	+	+	+
37	Caffeoylquinic lactone	11.41	-	-	-	tr
38	Isorhamnetin derivate	12.00	-	-	+	-
39	Myricetin-hexoside	12.38	-	-	+	-
40	Quercetin-pentoside-hexoside	12.84	+	+	+	tr
41	Quercetin-7-*O*-rutinoside	13.34	+	+	+	tr
42	Rutin (Quercetin-3-*O*-rutinoside)	13.67	+	+	+	+
43	Quercetin-glucoside	13.95	+	+	+	+
44	Unidentified	14.51	+	+	+	-
45	Unidentified	14.51	-	-	+	-
46	Unidentified	14.80	+	+	-	+
47	Kaempferol-3-*O*-rutinoside	15.05	+	+	+	+
48	3,4-Dicaffeoylquinic acid	15.19	+	+	tr	+
49	Kaempferol-3-*O*-glucoside	15.38	tr	-	tr	tr
50	Isorhamnetin-3-*O*-rutinoside	15.38	+	+	+	tr
51	3,5-Dicaffeoylquinic acid	15.46	+	+	tr	+
52	Isorhamnetin-3-*O*-glucoside	15.76	tr	+	+	tr
53	Dicaffeoylquinic acid	15.76	+	+	-	+
54	Isorhamnetin glucoside	16.10	-	-	+	-
55	Quercetin-rutinoside	16.25	-	-	+	-
56	Dicaffeoylquinic acid	16.31	tr	tr	-	+
57	4,5-dicaffeoylquinic acid	16.66	+	+	+	+
58	Hexoside derivate	16.95	+	+	+	-
59	Isorhamnetin 3-*O*-acetylglucoside	17.05	-	-	+	-
60	Hexoside derivate	17.13	tr	-	+	-
61	Hexoside derivate	17.49	tr	-	+	-
62	Caffeoylferuoyl-quinic acid	17.79	tr	tr	-	+
63	Caffeoylferuoyl-quinic acid	17.98	-	-	-	+
64	Caffeoylquinic acid	18.51	+	+	-	+
65	Caffeoylferuoyl-quinic acid	18.71	+	+	-	+
66	Caffeoylferuoyl-quinic acid	19.01	tr	-	-	+

Table legend: + component present;—component absent; * tentatively identyfied component (lack of accurate spectral literature database); tr—trace ions (deprotonated molecules) were observed; *I.PA*—yerba mate, *I. paraguariensis*; *I.AQ*—*I. aquifolium*; *I*.’*AM*’—*I. aquifolium* ‘Argentea Marginata’; *I.*×*M*—*I.* × *meserveae* ‘Blue Angel’.

**Table 3:** Quantification of polyphenols in *Ilex* leaves water extracts (average from three experiments).

No.	Component	RT	*I*.*AQ*	*I.*’*AM*’	*I.*×*M*	*I.PA*
	Caffeoylglucoses [mg ChE g^−1^] *					
6	Caffeoylglucose I	4.09	-	-	-	0.70 ± 0.00
14	Caffeoylglucose IV	6.47	-	-	-	1.15 ± 0.01
15	6-Caffeoylglucose	7.21	-	-	-	1.14 ± 0.01
	*p*-Coumaroylquinic acid derivate [mg pCE g^−1^] **					
16	3-*p*-Coumaroylquinic acid	7.49	0.23 ± 0.01	0.10 ± 0.00	-	-
27	4-*p*-Coumaroylquinic acid	10.23	0.12 ± 0.00	0.05 ± 0.00	-	0.06 ± 0.00
	Caffeoylquinic derivates [mg ChE g^−1^] *					
13	Neochlorogenic acid (5-CQA)	5.94	5.13 ± 0.03	4.53 ± 0.03	1.70 ± 0.01	19.71 ± 0.02
20	Chlorogenic acid (3-CQA)	8.30	23.29 ± 0.17	11.64 ± 0.11	16.68 ± 0.05	17.14 ± 0.06
23	Cryptochlorogenic acid (4-CQA)	8.71	5.33 ± 0.01	4.29 ± 0.01	2.23 ± 0.02	11.70 ± 0.57
48	3,4-Dicaffeoylquinic acid	15.19	1.41 ± 0.00	2.49 ± 0.04	-	4.80 ± 0.01
51	3,5-Dicaffeoylquinic acid	15.46	2.43 ± 0.00	3.48 ± 0.01	-	13.27 ± 0.07
57	4,5-Dicaffeoylquinic acid	16.66	2.39 ± 0.00	3.53 ± 0.05	0.90 ± 0.00	8.85 ± 0.04
	Feruloylquinic derivates [mg ChE g^−1^] *					
22	3-Ferruoylquinic acid	8.63	0.72 ± 0.00	0.81 ± 0.00	-	0.63 ± 0.00
34	4- Ferruoylquinic acid	11.2	0.67 ± 0.01	0.63 ± 0.00	0.68 ± 0.01	0.80 ± 0.01
36	5- Ferruoylquinic acid	11.31	0.99 ± 0.02	0.77 ± 0.01	0.77 ± 0.01	0.89 ± 0.02
	Flavonoids [mg RuE g^−1^] ***					
24	Quercetin-di-glucoside	9.62	-	-	0.26 ± 0.01	-
25	Quercetin-deoxyhexoside-hexoside	9.81	-	-	1.13 ± 0.01	-
28	Flavonoid-derivate	10.51	-	-	0.23 ± 0.00	-
30	Kaempferol-rutinoside-hexoside	10.86	-	-	0.27 ± 0.00	-
33	* Isorhamnetin-3-*O*-gentiobioside	11.06	-	-	0.31 ± 0.01	-
35	* Isorhamnetin-rutinoside-glucoside	11.21	-	-	0.46 ± 0.01	-
38	Isorhamnetin derivate	12.00	-	-	0.25 ± 0.00	-
39	Myricetin-hexoside	12.38	-	-	0.18 ± 0.00	-
40	Quercetin-pentoside-hexoside	12.84	0.59 ± 0.01	0.47 ± 0.00	0.47 ± 0.00	-
41	Quercetin-7-*O*-rutinoside	13.34	0.26 ± 0.00	0.26 ± 0.00	0.20 ± 0.00	-
42	Rutin	13.67	4.25 ± 0.02	4.37 ± 0.03	2.21 ± 0.01	2.88 ± 0.02
43	Quercetin-glucoside	13.95	0.47 ± 0.00	0.50 ± 0.00	0.46 ± 0.01	0.51 ± 0.02
47	Kaempferol-3-*O*-rutinoside	15.05	0.20 ± 0.00	-	0.38 ± 0.01	0.23 ± 0.01
49	Isorhamnetin-3-*O*-rutinoside	15.38	-	-	0.87 ± 0.12	-
52	Isorhamnetin-3-*O*-glucoside	15.76	-	0.23 ± 0.00	0.30 ± 0.00	-
59	Isorhamnetin 3-*O*-acetylglucoside	17.05	-	0.17 ± 0.00	0.28 ± 0.01	-
Sum of hydroxycinnamic acids derivatives ****			81.31 ± 1.81	42.71 ± 0.19	32.31 ± 0.05	22.96 ± 0.03
Sum of flavonoids [mg RuE g^−1^] ***			3.91 ± 0.09	5.78 ± 0.00	6.00 ± 0.02	8.26 ± 0.16

Table legend: *I.PA*—yerba mate, *I. paraguariensis*; *I.AQ*—*I. aquifolium*; *I*.’*AM*’—*I. aquifolium* ‘Argentea Marginata’; *I.*×*M*—*I.* × *meserveae* ‘Blue Angel’; * [mg ChE g^−1^]—concertation as chlorogenic acid equivalent (mg) per g of herb; ** [mg pCE g^−1^]—concertation as p-coumaric acid equivalent (mg) per g of herb; *** [mg RuE g^−1^]—concertation in rutin equivalent (mg) per g of herb; - = -components absent or too low concertation to quantification; **** Sum of hydroxycinnamic acids derivatives was calculated as sum of mg [ChE g^−1^] and mg [pCE g^−1^].

**Table 4:** Minimum inhibitory concentration (MIC) and minimum bactericidal/fungicidal concentration (MBC/MFC *) of *Ilex* water extracts (average from three experiments).

Extract	MIC & MBC */MFC * (mg mL^−1^)					
	*Escherichia coli* ATCC 10536	*Staphylococcus aureus* DSM799	*Candida albicans* DSM1386	*Alternaria alternata* CBS1526	*Fusariumoxysporum* KB-F1	*Aspergillus niger* DSM1957
*I.AQ*	0.51 0.51 *	0.26 0.26 *	1.02 >2.06 *	1.02 2.06 *	2.06 >2.06*	2.06 >2.06 *
*I.’AM’*	1.02 2.06 *	0.51 1.02 *	2.06 >2.06 *	1.02 2.06 *	2.06 >2.06 *	2.06 >2.06 *
*I.*×*M*	1.02 2.06 *	1.02 2.06 *	2.06 >2.06 *	2.06 >2.06 *	1.02 2.06 *	2.06 >2.06 *
*I.PA*	1.02 2.06 *	1.02 2.06 *	2.06 >2.06 *	2.06 2.06 *	2.06 2.06 *	2.06 >2.06 *

Legend: *I.PA*—*I. paraguariensis*; *I.AQ*—*I. aquifolium*; *I.’AM’*—*I. aquifolium* ‘Argentea Marginata’; *I.×M*—*I.* × *meserveae* ‘Blue Angel’; * values of minimal bactericidal (MBC) or fungicidal concentration (MFC).

## Data Availability

Research data is available from authors.

## Sample Availability

Samples of the compounds *I. paraguariensis*, *I. aquifolium* L., *I. aquifolium* ‘Argentea Marginata’ and *Ilex* × *meserveae* ‘Blue Angel’ are available from the authors.

## Appendix A

Appendix A contains additional table and figures about UHPLC-DAD-MS/MS analyses of *Ilex* samples. They are listed below:

**Table A1:** Expanded results of UHPLC-DAD-MS/MS analysis of analysed *Ilex* leaves extracts.

NB	Component	RT	UV λ_nm_ Max	MS ^1^ *m/z*	MS ^2^ Base Peak *m/z*	MS2 Secondary Peaks—*m/z* (Abundance)	[M−H]^−^ Formula	Error [Da]	Error [ppm]	Ref.
1	Quinic acid ^a,b,c^	0.92	258	191.0207	111.0087	-	C6H7O7	−0.9	−4.8	standard
2	Isomer of lithospermoside I ^b,c^	2.58	258	328.1101	130.0336	121.034 (20.16), 148.043 (19.10)	C14H18NO8	−0.6	−1.8	[19]
3	Isomer of lithospermoside II ^b,c^	2.75	259	328.1043	130.0305	121.0295 (40.06), 102.0358 (36.89), 125.0218 (3.17)	C14H18NO8	−0.6	−1.8	[19]
4	Unidentified	3.39	258	371.0984	191.0563	135.0455 (5.18), 161.0237 (3.29), 173.0456 (2.60)	C16H19O10	0.0	−0.1	-
5	* 3-Hydroxy-4-*O*-glucosylbenzoic acid ^b,c^	3.90	259	315.0720	108.0227	152.0121 (80.47)	C13H15O9	0.2	0.6	[20,21,22]
6	Caffeoylglucose I ^b,c^	4.09	326	341.0889	161.0264	135.0467 (22.08), 179.0353 (13.14)	C15H17O9	−1.1	−3.2	[8,22,23]
7	* Canthoside B ^b,c^	4.75	257	463.1454	233.0671	293.0870 (58.98), 125.0252 (50.23), 123.0448 (34.74), 149.0461 (32.35), 191.0550 (17.67), 131.0351 (10.15), 105.0348	C19H27O13	0.4	0.8	[24,25]
8	Caffeoylglucose II ^b,c^	4.81	327	341.0885	161.0266	135.0475 (55.38), 179.0356 (14.39), 221.051 (7.01)	C15H17O9	−0.9	−2.7	[8,22,23]
9	Theobromine ^a^	5.07	273	-	-	-	-	−	−	standard
10	Caffeoylglucose III ^b,c^	5.14	327	341.0878	161.0267	135.0473 (49.92), 179.0386 (18.17), 221.051 (3.04)	C15H17O9	−0.9	−2.7	[8,22,23]
11	* *p*-Hydroxybenzoic acid ^a,b,c^	5.50	280	137.0246	108.0238	136.0166 (75.91)	C7H5O3			[26]
12	*cis*-3-Caffeoylquinic acid ^b,c^	5.51	321	353.0886	191.0594	135.0474 (63.62), 179.0375 (38.53)	C16H17O9	−0.8	−2.2	[8]
13	Neochlorogenic acid (5-CQA) ^b,c^	5.94	324	353.0888	191.0586	135.0477 (69.82), 179.0377 (45.58), 161.0266 (5.09)	C16H17O9	−1.2	−3.3	[8]
14	Caffeoylglucose IV ^b,c^	6.47	326	341.0890	161.0266	135.0472 (24.92), 179.0381 (17.14), 203.0384 (3.96), 221.0474 (2.19)	C15H17O9	−1.2	−3.5	[8,22,23]
15	6-Caffeoylglucose ^b, c^	7.21	324	341.0890	161.0263	135.0481 (63.45), 179.0377 (63.45), 221.0475 (42.31), 281.0661 (5.44)	C15H17O9	−1.2	−3.5	[8,22,23]
16	3-*p*-Coumaroylquinic acid ^b,c^	7.46	313	337.0938	119.0519	163.0417 (82.87), 191.0586 (14.73)	C16H17O8	−0.9	−2.8	[8]
17	Caffeoylquinic acid ^b,c^	7.61	324	353.0882	135.0464	191.0570 (96.13), 173.0450 (80.28), 179.0354 (67.36), 93.0365 (10.89), 155.0352 (8.34), 111.0467 (8.22), 137.0241 (7.54)				[8]
18	Caffeoylglucose VI ^b,c^	8.05	324	341.0890	161.0268	135.0475 (80.13), 179.0367 (76.09) 221.0472 (44.08), 281.0662 (8.54), 203.0385 (2.33)	C15H17O9	−1.1	−3.4	[8,22,23]
19	Caffeic acid ^a, b, c^	8.17	321	179.0371	135.0473	117.0362 (6.09)	C9H7O4	−1.2	−3.5	standard
20	Chlorogenic acid (3-CQA) ^a,b,c^	8.30	324	353.0883	191.0584	161.0265 (2.08)	C16H17O9	0.7	1.9	standard
21	Caffeine ^a^	8.52	278	-	-	-	-			standard
22	3-Feruloylquinic acid ^b,c^	8.63	325	367.1036	134.0399	193.0528 (72.78), 149.0625 (10.02), 117.0361 (9.64)	C17H19O9	−0.1	−0.3	[8]
23	Cryptochlorogenic acid (4-CQA) ^b,c^	8.71	324	353.0892	135.0473	191.0583 (97.91), 179.0374 (81.84), 161.0269 (8.46),	C16H17O9	−1.4	−3.9	[8,9]
24	Quercetin-di-glucoside ^b,c^	9.62	352, 253	625.1411	625.1416	463.0865 (80.18), 301.0368 (15.78), 299.0190 (9.67)	C27H29O17	0.1	0.1	[27,28]
25	Quercetin-deoxyhexoside-hexoside ^b,c^	9.81	353, 255	609.1471	-	-	C27H29O16	−1.0	−1.6	[29]
26	*cis*-5-Caffeoylquinic acid ^b,c^	9.97	325	353.0878	191.0585	-	C16H17O9	−1.2	−3.5	[8]
27	4-*p*-Coumaroylquinic acid ^b,c^	10.23	312	337.0940	173.0478	191.058 (55.34), 119.0522 (37.71), 163.0418 (34.04), 137.0269 (13.06)	C16H17O8	−1.4	−3.9	[8]
28	* Flavonoid-derivate ^b,c^	10.51	353, 255	667.1523	-	-	C29H31O18	−0.7	−1	[29]
29	Kaempferol-di-hexoside ^b,c^	10.66	-	609.1463	447.0917	285.0413 (73.38), 609.1441 (41.45), 283.0255 (24.85), 327.0522 (8.38), 489.0952 (4.21)	C27H29O16	−0.2	−0.3	[28]
30	Kaempferol-rutinoside-hexoside ^b,c^	10.86	346, 265	755.2039	593.1502	755.2028 (25.24), 447.0918 (4.80), 285.0397 (4.05)	C33H39O20	0.2	0.2	[30,31]
31	Unidentified	10.92	282	537.1980	375.1456	357.1344 (33.02), 537.1990 (32.01), 489.2040 (25.25), 119.0356 (21.60), 327.1211 (19.19), 321.1131 (17.17), 339.1247	C26H33O12	−0.3	−0.5	-
32	Unidentified	11.06	-	593.1519	593.1487	473.1092 (30.56), 353.0676 (16.77), 383.0765 (7.87), 503.1181	C27H29O15	−0.7	−0.1	-
33	* Isorhamnetin-3-*O*-gentiobioside ^b,c^	11.06	345, 254	639.1576	477.1040	315.0504 (61.04), 639.1571 (28.77), 313.0387 (12.37)	C28H31O17	−1.0	−1.6	[32,33]
34	4-Feruloylquinic acid ^b,c^	11.20	324	367.1045	173.0476	134.0394 (41.39), 193.0527 (22.24)	C17H19O9	−1.0	−2.8	[8,9]
35	* Isorhamnetin-rutinoside-glucoside ^b,c^	11.21	352, 254	785.2152	623.1603	785.2078 (15.49), 315.0503 (6.79), 477.0992 (6.61)	C34H41O21	−0.7	−0.8	[32,33]
36	5-Feruloylquinic acid ^b,c^	11.31	325	367.1046	191.0583	134.0403 (24.8), 193.0538 (23.18),173.0476 (16.98), 149.0632 (4.52)	C17H19O9	−1.2	−3.2	[8,9]
37	Caffeoylquinic lactone ^b,c^	11.41	-	335.0787	-	-		−1.5	−3.5	[8]
38	Isorhamnetin derivate ^b,c^	12.00	349, 253	725.1572	519.1137	681.1659 (63.20) 477.1007 (27.15) 315.0503 (16.33), 561.1252 (13.86), 357.0587 (5.06)	C31H33O20	−0.1	−0.2	-
39	Myricetin-hexoside ^b,c^	12.38	355, 254	479.1206	317.0683	299.0591 (15.88), 479.1203 (13.93), 289.0719 (11.26) 0.001640, 165.0210	C22H23O12	−1.1	−2.4	[34]
40	Quercetin-pentoside-hexoside ^b,c^	12.84	352, 256	595.1310	300.0282	595.1303 (55.26), 179.0017 (2.11)	C26H27O16	−0.5	−0.9	[35,36]
41	Quercetin-7-O-rutinoside ^b,c^	13.34	353, 256	609.1457	609.1456	300.0291 (47.5)	C27H29O16	1.5	2.3	[29]
42	Rutin (Quercetin-3-*O*-rutinoside) ^a,b,c^	13.67	353, 256	609.1445	609.1441	300.0312 (43.16)	C27H29O16	1.6	2.6	standard
43	Quercetin-glucoside ^b,c^	13.95	352, 256	463.0878	300.0301	-	C21H19O12	0.4	0.9	[9,29]
44	Unidentified	14.51	392, 263	701.1363	701.1355	335.0193 (67.64), 509.0718 (9.63), 191.0564 (4.69), 669.0322 (2.02)	C32H29O18	−0.3	−0.05	-
45	Unidentified	14.51	385, 251	493.1338	331.0837	-	C23H25O12	−1.8	−3.7	-
46	Flavonoid ^c^	14.80	354, 257	547.2386	149.0481	191.0585 (30.48), 131.0365 (15.69), 101.0264 (11.61)	C25H39O13	1.0	1.9	-
47	Kaempferol-3-*O*-rutinoside ^b,c^	15.05	348, 265	593.1495	285.0415	-	C27H29O15	1.7	2.9	[8,9,37]
48	3,4-Dicaffeoylquinic acid ^b,c^	15.19	325	515.1185	173.0477	179.0372 (99.12), 353.0887 (54.25), 191.0583 (46.01), 161.0262 (19.59), 135.0474 (16.21)	C25H23O12	1.0	2.0	[8,9]
49	Kaempferol-3-*O*-glucoside ^b,c^	15.38	345, 254	447.0928	284.0331	447.0938 (14.96), 255.0305 (12.35), 227.0358 (6.66)	C21H19O11	0.5	1.0	[8,38]
50	Isorhamnetin-3-*O*-rutinoside ^b,c^	15.38	353	623.1621	315.0518	623.1618 (90.32)	C28H31O16	−0.4	−0.6	[39]
51	3,5-Dicaffeoylquinic acid ^b,c^	15.46	326	515.1184	191.058	179.0371 (66.61), 353.0882 (45.97), 135.0472 (11.41), 161.0263 (4.97)	C25H23O12	1.1	2.1	[8,9]
52	Isorhamnetin-3-*O*-glucoside ^b,c^	15.76	353, 255	477.1032	314.0442	477.1005 (36.22), 286.0504 (8.18), 299.0195 (4.73)	C22H21O12	0.7	1.5	[33,40]
53	Dicaffeoylquinic acid ^b^	15.76	325	515.1204	191.0566	179.0358 (73.65), 353.0878 (30.52), 173.0459 (16.28), 135.0447 (11.64), 161.0257 (11.13), 335.0779 (5.08), 359.0566 (2.56), 155.0362 (2.03)	C25H23O12	−0.7	−1.4	[8]
54	Isorhamnetin glucoside ^b,c^	16.10	253	477.1051	314.0431	299.0193 (93.25), 477.1052 (51.68), 271.0265 (10.28)	C22H21O12	−1.2	−2.5	[40,41]
55	Quercetin-rutinoside ^b,c^	16.25	284	609.1829	301.0717	609.1807 (5.26)	C28H33O15			[29]
56	Dicaffeoylquinic acid ^b,c^	16.31	325	515.1197	173.0458	179.0358 (84.27), 707.1842 (54.47), 191.0560 (38.01) 161.0247 (13.78), 135.0454 (10.60), 354.0899 (8.68), 155.0340 (4.89), 335.0758 (2.88), 163.0756 (2.37), 243.0645 (2.37)	C25H23O12	−0.2	−0.5	[8]
57	4,5-dicaffeoylquinic acid ^b,c^	16.66	326	515.1188	173.0479	179.0373 (77.99), 353.0885 (65.03), 191.0586 (30.17), 135.0477 (10.58), 161.0271 (2.79)	C25H23O12	0.7	1.4	[8,9]
58	* Hexoside derivate ^c^	16.95	-	431.1917	207.1381	251.1300 (94.96), 113.0225 (16.89), 335.0227 (15.87), 119.0341, 331 (11.20), 99.0457 (10.80), 101.0234 (9.81), 163.1470 (2.64)	C20H31O10	0.5	1.3	-
59	Isorhamnetin 3-*O*-acetylglucoside	17.05	351	519.1147	314.0451	299.0217 (15.41), 519.1172 (6.32)	C24H23O13	−0.1	−0.2	[41,42]
60	* Hexoside derivate ^c^	17.13	310	429.1771	205.1236	249.1115 (74.12), 161.1358 (44.20), 99.0439 (9.41), 153.0933 (3.39)	C20H29O10	−0.5	−1.1	-
61	* Hexoside derivate ^c^	17.49	-	431.1919	207.1392	251.1292 (81.26), 99.0452 (15.10), 113.0234 (10.61), 189.1290 (9.72), 119.0352 (9.70), 101.0266	C20H31O10	0.7	1.4	-
62	Caffeoylferuoyl-quinic acid ^b^	17.79	326	529.1342	193.0529	367.1029 (17.65), 173.0467 (6.66), 134.0391 (3.39), 191.0622 (2.19)	C26H25O12	1.0	1.8	[8]
63	Caffeoylferuoyl-quinic acid ^b^	17.98	326	529.1322	191.0583	179.0366 (44.92), 353.0898 (14.56), 173.0496 (12.08), 193.0512 (11.45), 135.0465 (10.73), 367.1057 (10.36) 161.0259 (8.01)	C26H25O12	2.0	4.0	[8]
64	Caffeoylquinic acid ^b,c^	18.51	326	515.1176	173.0479	179.0374 (90.57), 353.0892 (58.97), 191.0573 (45.39), 135.0479 (13.9), 161.0254 (13.9 5.49)	C25H23O12	1.9	3.7	[8]
65	Caffeoylferuoyl-quinic acid ^b^	18.71	325	529.1338	173.0478	193.0519 (21.1), 367.1043 (19.77), 137.0254 (2.43)	C26H25O12	1.3	2.5	[8]
66	Caffeoylferuoyl-quinic acid ^b^	19.01	-	529.1322	173.0475	179.0368 (79.05), 191.057 (73.88), 353.0882 (52.19), 135.0483 (10.6), 367.103 (9.32), 161.0303 (6.47) 137.0279 (4.76)	C26H25O12	1.9	3.7	[8]

Table legend; NB—number; RT—retention time; [M–H+]^−^ formula—calculated experimental deprotonated molecular formula; * tentatively identyfied component (lack of accurate spectral literature database; suspected isomer of a known component or unclear UV spectrum); ^a^ component identified by comparison with a standard; ^b^ component identified by comparison of UV and/or MS/MS spectra with literature; ^c^ component identified by prediction with MS/MS spectra;—lack of experimental UV spectra/ions or not applicable.
